# ISIEA: An image database of social inclusion and exclusion in young Asian adults

**DOI:** 10.3758/s13428-021-01736-w

**Published:** 2021-12-16

**Authors:** Zixin Zheng, Sijin Li, Licheng Mo, Weimao Chen, Dandan Zhang

**Affiliations:** 1 School of Psychology, Shenzhen University, Shenzhen, 518060 China; 2 Magnetic Resonance Imaging (MRI) Center, Shenzhen University, Shenzhen, 518060 China; 3 Shenzhen-Hong Kong Institute of Brain Science, Shenzhen, 518055 China

**Keywords:** Social exclusion, Social inclusion, Ostracism, Image database, Social cognition, Social reward, Social punishment

## Abstract

Human beings have a fundamental need to belong. Evaluating and dealing with social exclusion and social inclusion events, which represent negative and positive social interactions, respectively, are closely linked to our physical and mental health. In addition to traditional paradigms that simulate scenarios of social interaction, images are utilized as effective visual stimuli for research on socio-emotional processing and regulation. Since the current mainstream emotional image database lacks social stimuli based on a specific social context, we introduced an open-access image database of social inclusion/exclusion in young Asian adults (ISIEA). This database contains a set of 164 images depicting social interaction scenarios under three categories of social contexts (social exclusion, social neutral, and social inclusion). All images were normatively rated on valence, arousal, inclusion score, and vicarious feeling by 150 participants in Study 1. We additionally examined the relationships between image ratings and the potential factors influencing ratings. The importance of facial expression and social context in the image rating of ISIEA was examined in Study 2. We believe that this database allows researchers to select appropriate materials for socially related studies and to flexibly conduct experimental control.

**Supplementary Information:**

The online version contains supplementary material available at 10.3758/s13428-021-01736-w.

## Introduction

Complex social interactions pervade our daily lives because of human beings’ social nature. As a fundamental human need, the need to belong strongly drives people’s desire to establish and maintain interpersonal relationships with others (Baumeister & Leary, 1995). Whether belonging needs are satisfied has a significant impact on our physical and mental health (Baumeister & Leary, 1995). Positive social ties, such as being socially included, accepted, or supported, have been widely observed to be associated with pleasant feelings, healthy physiological functions, and good physical health (Kiecolt-Glaser et al., 2010; Eisenberger, 2003, 2013; Pressman, 2019). Some researchers have suggested that positive emotions, positive social connections, and physical health influence each other in a self-sustaining upward-spiral dynamic (Kok et al., 2013). Conversely, social pain refers to a painful experience associated with actual or potential damage to desired social connections (Eisenberger, 2012, 2015), usually caused by social exclusion or rejection. Previous studies have found that social exclusion induces negative or painful emotions and threatens a human’s fundamental needs (Williams, 2007).

Traditional studies on social exclusion/inclusion generally use paradigms, such as cyberball (Williams et al., 2000; 2003), to simulate a social interaction scenario. The image-based imagination paradigm also serves as an effective way to examine the social interaction context (Elliott et al., 2012; Zhao et al., 2021). Humans can experience the empathic feeling of others' emotions by simulating what is happening in the minds of who they are observing (Singer & Lamm, 2009; Keysers & Gazzola, 2007). Studies have demonstrated that participants have similar behavioral and neural responses observed in the cyberball game when they view images of social exclusion situations (e.g., the activation of dorsal anterior cingulate cortex; Kross et al., 2007; Premkumar et al., 2012; Eisenberger, 2015). Meanwhile, other studies have found that simply viewing images of social support figures (e.g., romantic partners) can reduce painful experiences, supported by both self-reported and neural activity data (Eisenberger et al., 2011; Younger et al., 2010). Importantly, in a study investigating the neuronal deficits of social stimuli processing in individuals with major depression, Elliott et al. (2012) collected a set of images of social exclusion and inclusion scenarios and asked participants to imagine themselves as the highlighted person in the picture and to experience their affective feelings. Inspired by this study, our lab has performed a series of studies, using a set of exclusion images and asked participants to imagine feeling the emotions of the highlighted person in the images (He et al., 2018; 2020a, b; Zhao et al., 2021). These images were verified to be effective in inducing social pain, which helped us study the emotional regulation effect in response to social pain.

Although the image-based imagination paradigm may not elicit emotions as real as “first-hand” pain-inducing paradigms, such as the cyberball game, it contributes to social cognition research with its own advantages. Using images as stimuli is especially suitable for event-related designed neuroscience experiments that require precise time accuracy and an adequate number of trials for reliable signals (e.g., fMRI signals and event-related potentials). Moreover, image viewing is a simple and understandable task for specific participants, such as children and psychiatric patients, and this paradigm does not elicit excessively negative emotions that might hurt these participants (Singer & Lamm, 2009). Given these features and empirical evidence supporting the validity of the imagination paradigm and social image stimuli, a set of standardized images is urgently needed for research and clinical practice.

Images are widely used as effective visual stimuli in affective research. To date, there have been many emotional-image databases with ratings of emotional characteristics (Barrett, 2006; Dalgleish, 2004; Ekman, 1992). The most popular databases include the International Affective Image System (IAPS; Lang et al., 1999), the Geneva Affective Image Database (GAPED; Dan-Glauser & Scherer, 2011), and the Nencki Affective Image System (NAPS; Marchewka et al., 2014). These databases covered various themes of images, including people, animals, objects, and natural scenes. However, limited image databases have been identified in the field of social interactions. Some image databases provide social stimuli using isolated faces without social contextual information (e.g., the Chicago Face Database; Ma et al., 2015). The Pictures with Social Context and Emotional Scenes (PiSCES; Teh et al., 2018) is a valuable database that provides black-and-white line drawings depicting various social situations. However, it lacks a detailed classification of complex social situations, so it could not meet the need to examine specific social conditions, such as social inclusion and exclusion. Additionally, we think that line drawings decrease the realness and ecological validity of social interaction scenarios. Besides these databases, some studies used social inclusion/exclusion images collected from the internet (e.g., Elliott et al. (2012) used eight negative and eight positive social interaction images), but these materials are usually not open-access due to issues such as copyrights and the ratings of the emotional characteristics of each image were not provided. Due to limited image databases in the field of social interaction, especially social inclusion/exclusion, researchers must perform time-consuming additional work to collect or create materials themselves, which leads to the uncertain quality of materials, potential problems with copyright controversy, and a lack of comparability and generalization of experimental results.

Furthermore, most of these databases or study materials presented people/faces from Western countries, which may introduce confounding factors for Asian studies due to cultural differences (Boiger & Mesquita, 2012). For example, it was found that Turkish and Indian participants (collectivist background) showed different responses to social exclusion compared to German and American participants (individualist background) (Pfundmair et al., 2015), possibly because people from collectivist backgrounds perceive social exclusion as less threatening so they are less affected by it than people from individualistic backgrounds (Pfundmair et al., 2018). The influence of culture is also reflected in the presence of “in-group advantage,” i.e., individuals have stronger reactions to or better recognition of emotional information conveyed by individuals from the same race (Elfenbein & Ambady, 2002; Brown et al., 2006). This in-group advantage has also been found in the emotional processing of social events such as social exclusion (He et al., 2021; Krill & Platek, 2009). Considering the growing number of social-related studies from Asia, there is an urgent need for a standardized socioemotional image database.

This two-part research aims to create a standardized, open-access image database: the social inclusion/exclusion in young Asian adults (ISIEA) database, which displays three categories of social interaction contexts (social exclusion, social neutral, and social inclusion). Study 1 aims to establish and assess characteristics of ISIEA. Study 2 aims to explore the effect of image components (face and context) on the rating of the entire image.

## Study 1

This study conducts a standardized assessment of images and provides ratings of valence, arousal, inclusion score, and vicarious feeling for each image. The ratings of valence (ranging from unpleasant to pleasant) and arousal (ranging from calm to excited) have been widely applied as basic dimensions of emotion in many social and affective studies (Lang et al., 1999; Kuppens et al., 2013). The inclusion score represents the participant's perceived intensity of social exclusion or social inclusion from the scenario in the images (Elliott et al., 2012). Vicarious feelings reflect vicarious affective experiences when participants imagine themselves as the highlighted person in the image (e.g., being rejected). While the ratings of valence, arousal, and inclusion were reported by participants with a relatively safe distance from the images, the rating of vicarious feeling reflects a more engaged affective experience in the social scenario. Previous research has found that participants experienced a stronger need threat when they took the perspective of the person in the image who was being socially excluded (Wesselmann et al., 2009). For all rating scales, we utilized semantic bipolar scales (a nine-point Likert scale). Although this scale is different from the Self-Assessment Manikin scale utilized by the IAPS, it has been shown to be highly correlated with the SAM scale (Bradley & Lang, 1994) and has been adopted in many related studies (Kurdi et al., 2017; Marchewka et al., 2014; Teh et al., 2018). Since existing emotional image databases (e.g., IAPS; Lang et al., 1999) reveal that arousal and valence have an inverted U-shaped relationship, we thus hypothesized a similar quadratic relationship between arousal and valence/inclusion score/vicarious feelings. As an image database, the images’ physical properties, including luminance, contrast, complexity, and color features, are also provided.

### Materials and methods

#### Participants

A total of 63 healthy college students, including 37 females (22.8 ± 2.1 years old, mean ± standard deviation) and 26 males (23.2 ± 1.8 years old), were recruited from Shenzhen University to participate in the photographing as actors and actresses. For image ratings, we first recruited 100 healthy college students, including 50 females (20.1 ± 1.25 years old) and 50 males (20.7 ± 2.09 years old), from Shenzhen University to rate the images. Half a year after this initial sampling, an additional 50 healthy college students, comprising 21 females (22.8 ± 1.03 years old) and 29 males (22.0 ± 1.52 years old), were recruited to add the sample size. These two samples of image ratings were combined for data analyses because of their homogeneity. To enhance the sample diversity, all participants consisted of undergraduate and graduate students from various majors, including natural science, social science, engineering, and medicine. The study was approved by the Ethics Committee of Shenzhen University. Informed consent was signed by the participants before the photography or the image rating task. All the actors and actresses authorized the use of their portraits for the ISIEA database after they clearly understood the informed consent form. After photographing or picture ratings, participants were paid ¥50 as compensation.

#### Materials

A total of 164 images in the ISIEA were taken by our lab at Shenzhen University using a digital camera (Sony ILCE-7M2, Japan). To display various social situations on campus, we selected 104 outdoor scenes (playgrounds, school roads, lawns, etc.) and 60 indoor scenes (classrooms, libraries, offices, etc.) to enhance the diversity of the images’ backgrounds. All images can be downloaded freely from the website 10.5281/zenodo.5496421.

**Fig. 1:**
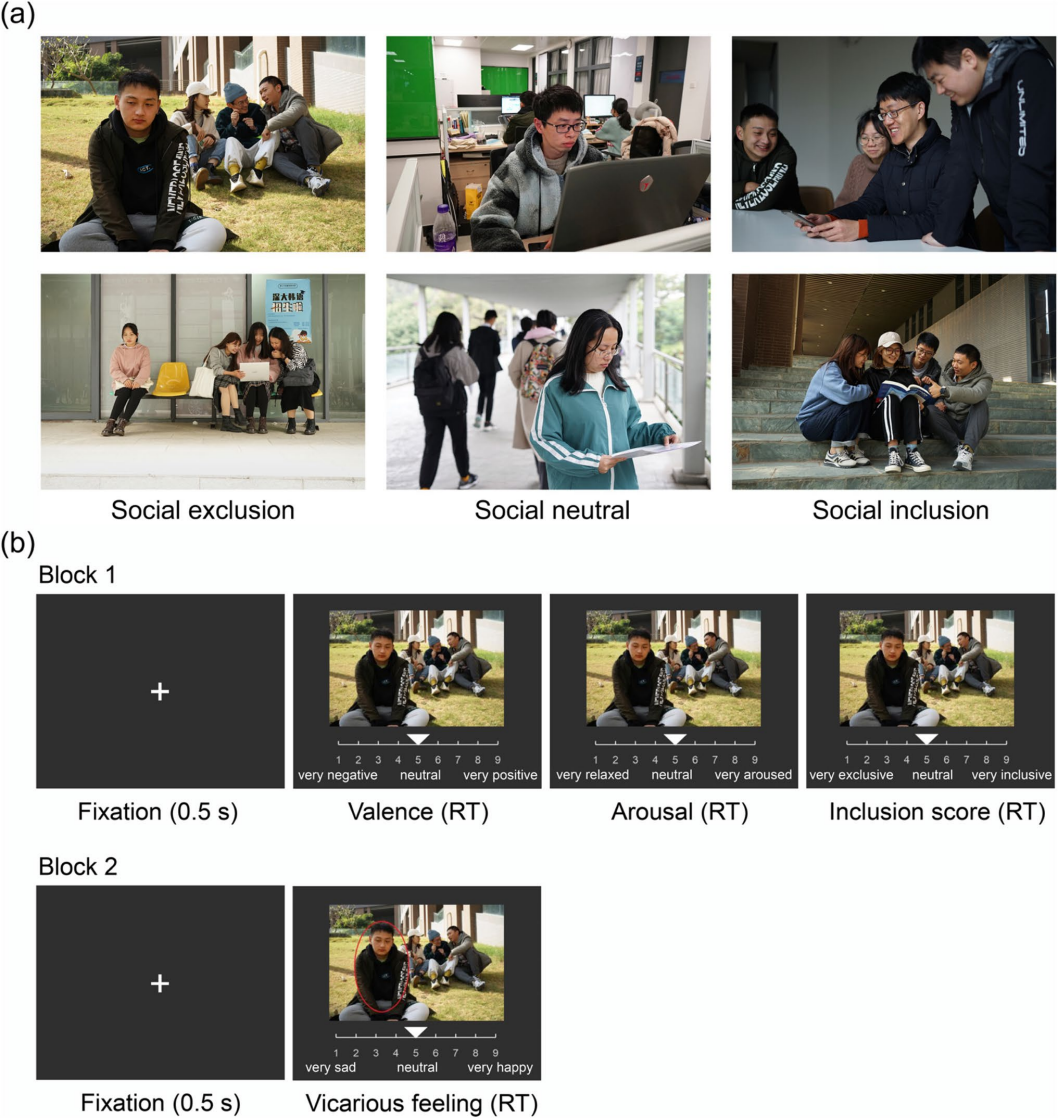
**a** Example pictures of social exclusion, social neutral, and social inclusion with female or male protagonists. **b** Trial design of the rating task. Each image remained on the screen until the participants finished their ratings (RT = reaction time). The instructions/labels were displayed in Chinese during the task and translated to English for the sake of publication. The font color is white, and the background was gray (RGB = [50, 50, 50])

As shown in Fig. 1a, there are three categories of images in the ISIEA. The “social exclusion” category (*N* = 60) depicts scenarios consisting of one rejectee with sad or upset facial and body expressions, and three or four rejecters talking and laughing together. The scenarios of the “social neutral” category (*N* = 53) are composed of two to five individuals with neutral expressions. No social interaction or communication existed in these images (i.e., individuals read books by themselves). The “social inclusion” category (*N* = 51) of images includes social scenarios of friendly communication between three or four individuals.

#### Physical properties of images

Physical parameters, such as luminance, contrast, complexity, and color parameters, were measured utilizing the criteria in an existing affective image database (Marchewka et al., 2014). We utilized the Image Processing Toolbox in MATLAB (MathWorks, Natick, MA) to compute these parameters. Luminance is the average pixel value of a grayscale image, and contrast is the standard deviation across all pixels of the grayscale image (Bex & Makous, 2002). The visual complexity of an image can be reflected by the JPG size and entropy. While the JPG size measures the overall complexity of an image (Donderi, 2006), entropy is a measure of randomness and reflects the texture of one image that was calculated using the MATLAB function *entropy.m* in this study. For the color parameters, we calculated the pixel mean of each channel based on the CIE L*a*b* color space. Compared with the RGB color space, this color space is based on the opponent-process theory of color vision, so it is closer to the characteristics of the human visual system. The L* channel represents the perceptual lightness ranging from 0 to 100; the a* channel represents the color spectrum range from green (negative values) to red (positive values), and the b* channel represents the color spectrum range from blue (negative values) to yellow (positive values). The physical properties of each image are presented in Supplementary Material. All images were the same size, 1200 × 800 pixels (300 dpi). Imaging software Photoshop (Adobe Systems Incorporated, San Jose, CA, USA) was used to adjust the color tone, brightness, and contrast of images to ensure that there was no difference in the measured physical characteristics across the three categories of images.

#### Picture rating

Images were presented sequentially in a randomized order using PsychoPy software (version 3.1.0; Peirce et al., 2019) on an LCD screen with a viewing angle of 3.0° × 3.5°. Prior to rating, we utilized oral and written instructions to ensure that participants were clear about the meanings of the terminologies used in the task.

The rating procedure consisted of two blocks (Fig. 1b). In the first block, participants judged the valence (from 1 = very negative, 5 = neutral, to 9 = very positive), then the arousal (from 1 = very relaxed, 5 = neutral, to 9 = very aroused), and finally the degree of social inclusion (from 1 = very exclusive, 5 = neutral, to 9 = very inclusive). We fixed the order of the three ratings for each image to avoid confusion and ensure that participants could rate valence, arousal, and inclusion level accurately (see also Teh et al., 2018). In the second block, all images appeared again, with a red circle highlighting one person in the image. Participants were instructed: “In this scenario, please think about how you would feel in a situation similar to that of the highlighted person in the image.” Participants were then asked to report this vicarious affective feeling (from 1 = very sad, 5 = neutral, to 9 = very happy) during image presentation. All ratings were reported by moving and clicking a bar on a horizontal scale using a computer mouse. Each image remained on the screen until participants finished their ratings. All 150 participants were presented twice with a complete set of 164 images in the two blocks. The entire rating procedure took approximately 40 min. Participants had a break of 2–3 min at the middle of each block, and a break of 10 min between two blocks. After the image rating task, participants were asked to complete self-reported questionnaires, including the Liebowitz Social Anxiety Scale (LSAS, Liebowitz, 1987) and the Interpersonal Reactivity Index (IRI, Davis, 1980).

#### Statistics

Statistical analysis was performed using SPSS Statistics (version 20.0; IBM, Somers, NY). Descriptive data are presented as mean ± standard deviation, unless otherwise stated. While we reported significant results in the main text for the sake of conciseness, a full report of statistical results could be found in Supplementary Material.

The ratings of valence, arousal, inclusion scores, and vicarious feelings were statistically analyzed. One-way ANOVAs were performed separately on each rating scale to compare the differences across the three categories of images. The Scheffe method was used to conduct a post hoc test (due to the unequal number of images across categories). Cronbach's α coefficient was used to measure the reliability of the image ratings across participants. Subsequently, the Pearson correlations between the ratings of valence, inclusion scores, and vicarious feelings were calculated. Owing to the quadratic relationship between arousal and valence, which has been widely reported in affective studies, we examined this relationship by fitting a quadratic regression. In addition, mixed design ANOVAs were performed to explore the time effect (details in Supplementary Material).

To explore the gender effect, 3 × 2 mixed design ANOVAs were performed separately for each rating scale, with the context category as the within-subject factor and gender as the between-subject factor (details in Supplementary Material). The potential influences of social anxiety and empathy of image raters were explored by conducting Pearson correlations between questionnaire scores and image ratings; multiple comparisons were corrected using the FDR method.

### Results

#### Ratings of valence, arousal, inclusion score, and vicarious feeling

**Fig. 2:**
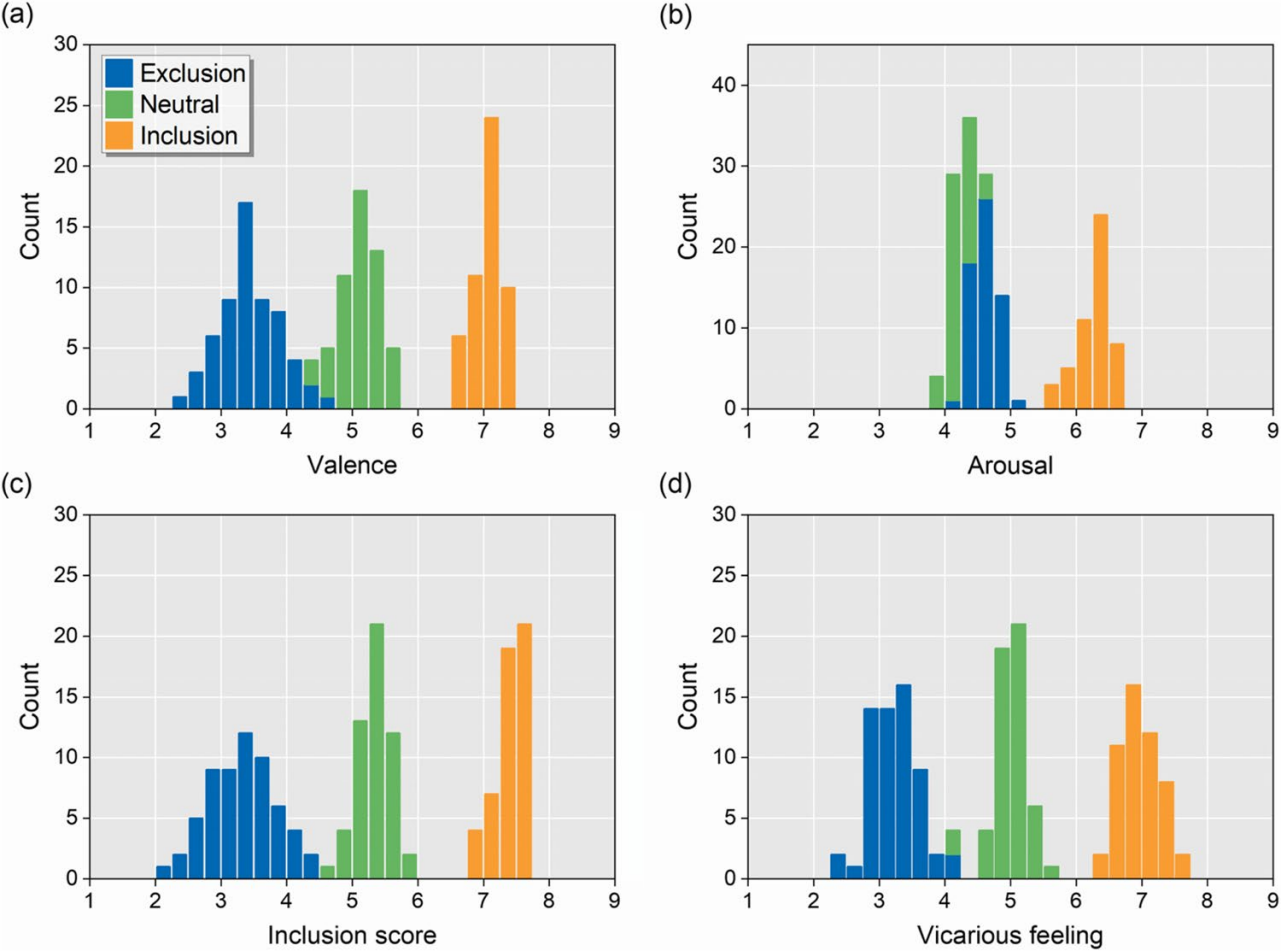
Distribution of image ratings for **a** valence, **b** arousal, **c** inclusion score, and **d** vicarious feeling. The colors correspond to different image categories

Ratings for each image are provided in Supplementary Material. The descriptive characteristics of the three categories are presented in Table 1. We also illustrate the rating distributions of the three categories of images in Fig. 2. The Kolmogorov–Smirnov test shows that the distributions of these ratings for the three categories of images conform to the normal distribution (all *p* ≥ .320, see Table S1 for exact statistics).

**Table 1:** Descriptive statistics of picture ratings in the three categories of images

Rating	Exclusion (*N* = 60)		Neutral (*N* = 53)		Inclusion (*N* = 51)	
	Mean ± SD	Range	Mean ± SD	Range	Mean ± SD	Range
Valance	3.428 ± 0.469	2.490–4.650	5.108 ± 0.290	4.420–5.680	7.064 ± 0.201	6.590–7.440
Arousal	4.590 ± 0.194	4.100–5.110	4.224 ± 0.162	3.940–4.720	6.269 ± 0.242	5.720–6.630
Inclusion score	3.312 ± 0.500	2.200–4.470	5.335 ± 0.257	4.650–5.810	7.395 ± 0.226	6.850–7.720
Vicarious feeling	3.214 ± 0.353	2.270–4.070	4.999 ± 0.263	4.070–5.590	6.968 ± 0.293	6.390–7.540

**Fig. 3:**
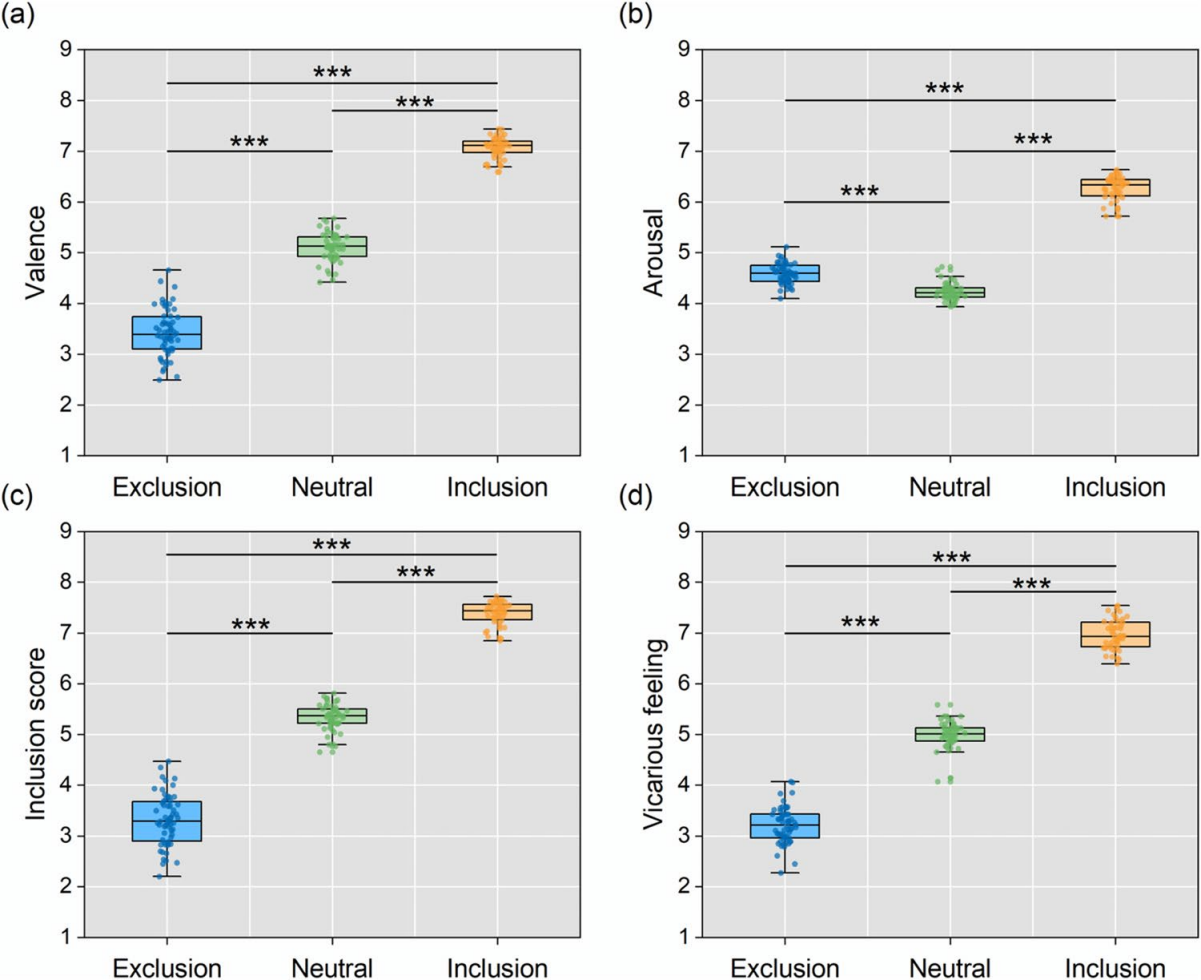
Picture ratings for the three categories for **a** valence, **b** arousal, **c** inclusion, and **d** vicarious feeling. Each *dot* represents the mean rating of an image; *boxes* represent the 25th and 75th percentiles, and the *whiskers* represent upper and lower values within 1.5 × inter-quartile range. Pairwise comparison: ***p < 0.001

One-way ANOVAs showed that the four ratings differed across the image categories (Fig. 3). For the valence of images, *F*(2,161) = 1516.2, *p* < .001, ${\upeta}_{\mathrm{p}}^2$= .950, participants rated social inclusion images as the most positive, followed by social neutral images, and social exclusion images were rated as the most negative (pairwise *p*s < .001). For the arousal rating, *F*(2,161) = 1537.9, *p* < .001, ${\upeta}_{\mathrm{p}}^2$= .950, participants reported the highest arousal for social inclusion images, followed by social exclusion images, and social neutral images were rated as having the lowest arousal (pairwise *p*s < .001). For the inclusion score, *F*(2,161) = 1787.5, *p* < .001, ${\upeta}_{\mathrm{p}}^2$= .957, social exclusion images were rated as having the lowest scores (indicating a high level of social exclusion), and social inclusion images were rated as having the highest scores, with social neutral images having median scores indicating neither exclusive nor inclusive (pairwise *p*s < .001). For the vicarious feeling, *F*(2,161) = 2052.0, *p* < .001, ${\upeta}_{\mathrm{p}}^2$= .962, participants reported the most positive feelings for social inclusion images, followed by social neutral images, and social exclusion images were associated with the most negative feelings (pairwise *p*s < .001).

We also conducted a reliability analysis for these four ratings to examine the consistency across participants. The results showed excellent internal consistency for all ratings (Table 2).

**Table 2:** Reliability (Cronbach's alpha) of picture ratings

Rating	Exclusion	Neutral	Inclusion
Valance	0.976	0.935	0.973
Arousal	0.984	0.984	0.987
Inclusion score	0.977	0.978	0.976
Vicarious feeling	0.978	0.906	0.961

#### Relationships between picture ratings

**Fig. 4:**
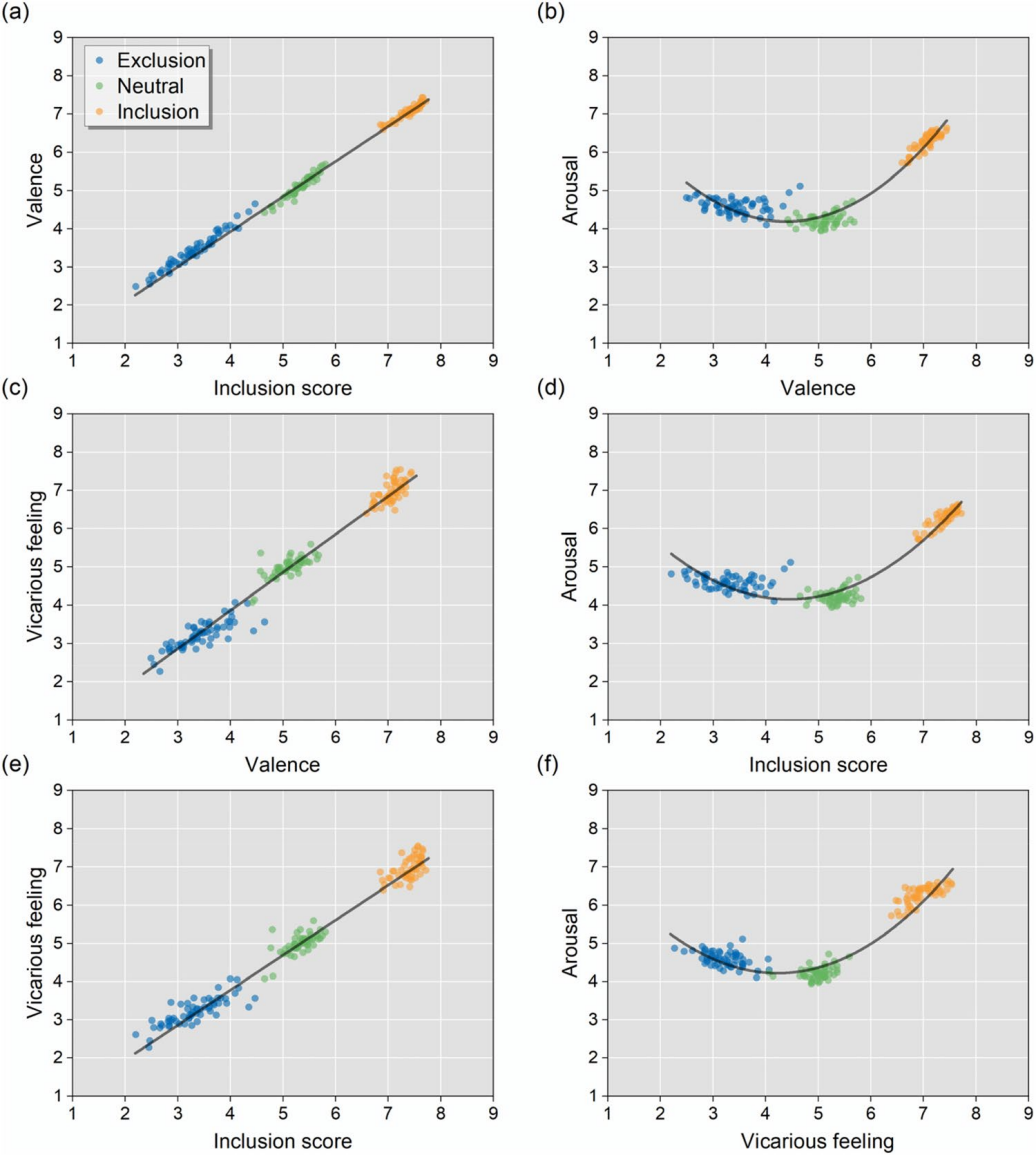
Relationships between **a** valence and inclusion score, **b** arousal and valence, **c** vicarious feeling and valence, **d** arousal and inclusion score, **e** vicarious feeling and inclusion score, and **f** arousal and vicarious feeling. Each *dot* represents the mean rating of an image. Colors correspond to different image categories

Correlation analysis showed a strong positive correlation between valence and inclusion score (*r* = .998), between valence and vicarious feeling (*r* = .988), and between inclusion score and vicarious feeling (*r* = .988, *p*s<.001; Fig. 4, panels a, c, e).

For the relationship between arousal and the other three ratings, quadratic regressions were performed (Table S2), and significant results were observed between arousal and valence (*R*^*2*^ = .936), inclusion score (*R*^2^ = .934), and vicarious feeling (*R*^2^ = .923, all *p*s < .001; Fig. 4, panels b, d, f). Generally, images with a high positive and negative valence or vicarious feelings, and images showing socially inclusive or exclusive scenarios were associated with high arousal scores.

#### Effect of gender, social anxiety, and empathy on picture ratings

The gender effect was examined utilizing two-way mixed design ANOVA (Table S4), which showed that neither the main effect of gender nor the interaction between gender and context was significant (all *p* ≥ .440, details in Supplementary Material). These results suggested that gender has no effect on images’ ratings.

Correlation analyses were performed between social anxiety/empathy and the four image ratings (Table S3). Results show that social anxiety was negatively correlated with valence (*r* = – .327, *p* < .001, *p*_*cor*_ < .001), inclusion score (*r* = – .193, *p* = .018, *p*_*cor*_ = .043), and vicarious feelings (*r* = – .324, *p* < .001, *p*_*cor*_ < .001) of social neutral images, which indicated that people with higher social anxiety tended to have more negative feelings in socially neutral scenarios. Social anxiety was also negatively correlated with vicarious feelings (*r* = – .199, *p* = .015, *p*_*cor*_ = .039), indicating people with high social anxiety have more negative feelings when vicariously experiencing social exclusion scenarios. Empathy was positively correlated with valence (*r* = .314, *p* < .001, *p*_*cor*_ < .001), inclusion score (*r* = .341, *p* < .001, *p*_*cor*_ < .001), and vicarious feeling (*r* = .287, *p* < .001, *p*_*cor*_ = .002) of social inclusion images, suggesting people with higher levels of empathy rate social inclusion images as more positive and more inclusive. Empathy was also negatively correlated with valence (*r* = – .255, *p* = .002, *p*_*cor*_ = .005), inclusion score (*r* = – .225, *p* = .006, *p*_*cor*_ = .017), and vicarious feeling (*r* = – .269, *p* < .001, *p*_*cor*_ = .003) of social exclusion images, indicating people with higher levels of empathy rate social exclusion images as more negative and more exclusive.

## Study 2

**Fig. 5:**
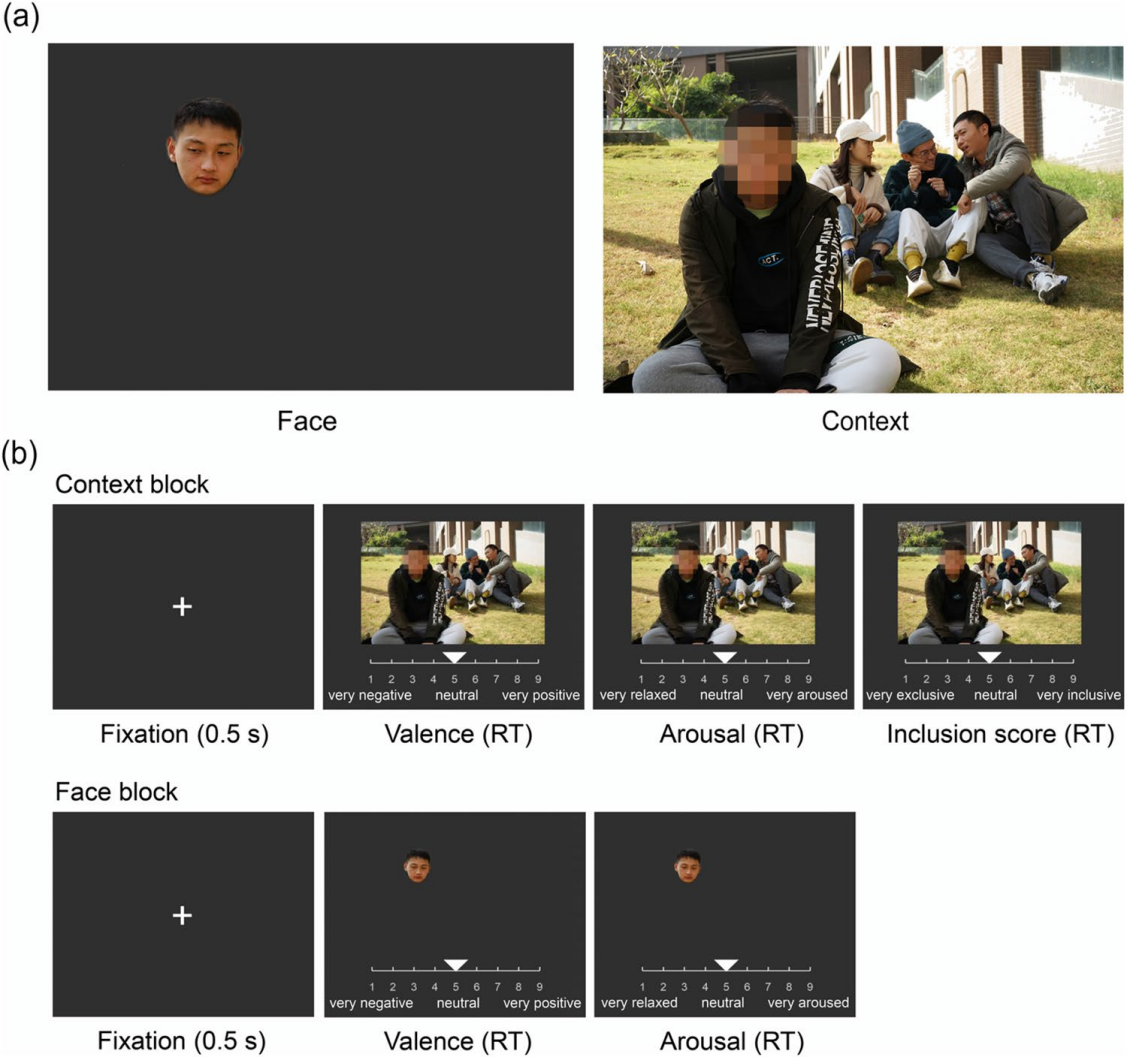
**a** Example pictures of face and context component. **b** The trial design of the rating task. Each image remained on the screen until the participants finished their ratings (RT = reaction time). The instructions/labels were displayed in Chinese during the task and translated to English for the sake of publication. The font color is white, and the background was gray (RGB = [50, 50, 50])

This study investigated whether image ratings were independently influenced by facial expressions or social contexts of images. Specifically, every image was segmented into face and context, forming two new sets of images (Fig. 5). Ratings for these two image sets were collected to examine the impact of facial expression and social context.

### Materials and methods

#### Participants

Another 50 healthy college students, comprising 24 females (21.1 ± 1.4 years old) and 26 males (21.8 ± 1.9 years old), were recruited from Shenzhen University. The participants samples of the two studies were homogeneous. Informed consent was signed before the image rating task. After image ratings, each participant was paid ¥50 as compensation.

#### Materials

For the face set, the face of the main character was extracted and placed on a gray background using Photoshop (Adobe Systems Incorporated, San Jose, CA, USA). For the context set, the face of the main character was masked by mosaic while the rest of the image was preserved as the context component. Both sets of images had the same size and resolution (1200 × 800 pixels, 300 dpi).

#### Picture rating

The rating task was divided into two blocks, corresponding to the face and context image sets. The order of blocks was counterbalanced across subjects. In the face block, participants judged the valence and arousal of each face image. In the context block, participants judged the valence, arousal, and inclusion levels of each context image. All the images were presented randomly with a fixed order of various ratings. The entire rating procedure took approximately 40 min. The rest setting was consistent with Study 1.

#### Statistics

Statistical analysis was performed using SPSS Statistics (version 20.0; IBM, Somers, NY, USA). Regression analyses were performed to investigate the influence of face/context ratings on the ratings of the entire image (enter method). First, two multiple regression models were fitted to predict the valence of the entire image using valence ratings of face and context as predictors, and to predict the arousal of the entire image using arousal ratings of face and context as predictors. Second, two multiple regression models were fitted to predict the inclusion score of the entire image using valence ratings of face and context as predictors, and to predict the inclusion score of the entire image using valence ratings of face and inclusion score of the context as predictors.

### Results

Regression analysis revealed that the ratings of both face and context images positively predicted the ratings of entire images (Table 3), indicating that the emotional attributes of face and context components independently affect the emotional attributes of the entire image. Descriptive statistics are reported in Table 4.

**Table 3:** Summary of regression analyses (*N* = 163)

Model	*β*	*β*_standardized_	t	*p*
(a) Dependent variable: valence of entire image R^2^ = .974, *F*(2,161) = 3024, *p* < .001				
Face valence	0.402	0.344	11.657	< .001
Context valence	0.779	0.665	22.505	< .001
(b) Dependent variable: arousal of entire image R^2^ = .961, *F*(2,161) = 1973, *p* < .001				
Face arousal	0.364	0.402	13.585	< .001
Context arousal	0.632	0.616	20.808	< .001
(c) Dependent variable: inclusion of entire image R^2^ = .969, *F*(1,161) = 2496, *p* < .001				
Face valence	0.369	0.283	8.713	< .001
Context valence	0.946	0.722	22.252	< .001
(d) Dependent variable: inclusion of entire image R^2^ = .967, *F*(1,161) = 3933, *p* < .001				
Face valence	0.337	0.258	10.087	< .001
Context inclusion scores	0.781	0.751	29.345	< .001

**Table 4:** Descriptive statistics of picture ratings for face, context, and the entire image

Component	Exclusion		Neutral		Inclusion	
	Valence	Arousal	Valence	Arousal	Valence	Arousal
Face	3.85 ± 0.49	3.96 ± 0.26	4.80 ± 0.55	4.04 ± 0.38	6.79 ± 0.39	5.97 ± 0.48
Context	3.82 ± 0.59	4.75 ± 0.23	5.21 ± 0.28	4.18 ± 0.28	6.83 ± 0.33	6.18 ± 0.37
Entire	3.43 ± 0.47	4.59 ± 0.19	5.11 ± 0.29	4.22 ± 0.16	7.06 ± 0.20	6.27 ± 0.24

### Discussion

Introducing the ISIEA database and its utilization in this study has shown promise. The ratings and physical parameters provided for these pictures in color (Supplementary Material) would be helpful for future image selection and control of confounding factors.

Category comparisons using ANOVA demonstrated significant differences in image ratings across socially inclusive, neutral, and exclusive images. Social exclusion images were rated as the most negative (lowest valence) and the most socially exclusive (lowest inclusion score), and evoked the most negative feelings (lowest vicarious feeling). The social inclusion images were rated as the most positive (highest valence) and the most socially inclusive (highest inclusion score), and evoked the most positive feelings (highest vicarious feeling). The social neutral images were rated as neither negative nor positive (medium valence), neither exclusive nor inclusive (medium inclusion score), and evoked neutral feelings (medium vicarious feeling). Regarding the arousal rating, the highest scores were obtained for social inclusion images, possibly due to more explicit social interactions with intense facial expressions (i.e., laughter) or obvious body language (i.e., waving hand). Meanwhile, social exclusion images had stronger arousal than social neutral images, although the difference between these two categories was smaller due to the relatively low arousal of social exclusion images (individuals usually displayed sad, upset, or calm facial and body expressions). The high Cronbach’s α coefficients for the four ratings indicated high agreement among participants on the emotional experience and interpretation of social content in each context category.

Regarding the relationship between these picture ratings, the strong positive correlation between valence, inclusion score, and vicarious feeling suggest that the valence and vicarious affective feelings are highly related to the level of social exclusion/inclusion that participants perceived from the scenarios. The relationship between valence and arousal was quadratic. This typical boomerang shape between valence and arousal has been widely reported in many databases (Kurdi et al., 2017; Lang et al., 1999; Marchewka et al., 2014). Likewise, a quadratic relationship was found between arousal and inclusion score/vicarious feeling, indicating that images with high social inclusion and exclusion levels and images perceived as having strong positive and negative vicarious emotions are more arousing than images with a neutral social context.

We also explored the potential influential effects on image ratings. First, individuals’ social anxiety magnified the negative feelings they experienced from socially neutral and exclusion images. This is consistent with previous studies reporting that socially anxious individuals tend to negatively interpret ambiguous social information (Mobini et al., 2013; Peschard & Philippot, 2017), and that they are sensitive and show strong negative emotional responses to various events that cause social distress (Auyeung & Alden, 2016; Goldin et al., 2009; Harb et al., 2002). Second, we found that a high capacity for empathy enhanced participants’ emotional experience toward exclusion and inclusion images. The ability of empathy is the cornerstone of social function, enabling us to understand others’ mental and affective states (Singer & Lamm, 2009). Numerous studies have shown that observed and imagined experiences of social exclusion shared similar emotional experiences and neural responses (Eisenberger, 2012; Giesen & Echterhoff, 2018; Meyer et al., 2013; Wesselmann et al., 2009). For instance, Masten et al. (2011) found that trait empathy is positively correlated with the neural activity of empathy-related brain regions when participants experienced social exclusion displayed in images, suggesting that the emotional experience induced by exclusion images is significantly influenced by empathic ability. In general, these findings suggest that researchers should consider individual differences when employing our image database in their studies.

In Study 2, we examined the role of facial expression and social context in the emotional rating of ISIEA. While isolated facial expressions usually transfer clear basic emotions, their interpretation becomes more complicated in socially interactive contexts. For example, facial expressions of laughter and frustration may coexist in scenes of social exclusion. In these cases, contextual information that helps us accurately perceive and interpret others’ emotions is critical (Olsson & Ochsner, 2008). Our results demonstrated that the emotional attributes of both facial expression and social context contribute to the ratings of an entire image, suggesting that social context is essential for delivering inclusion/exclusion information.

Furthermore, we suggest considering the following aspects when using images from this database. First, the social exclusion images were produced according to a narrow definition of social exclusion or ostracism (Williams, 2007) that did not include images displaying overt abuse, finger-pointing reproach, or other strong negative facial expressions (e.g., crying and fear). This is also the reason for the lack of high-arousal images in the social exclusion category. Second, while this database contains images of and is rated by young adults, people of different ages may have different interpretations, emotional experiences, and neural responses to context-based social images. For instance, a relevant meta-analysis study found that adults mainly activated the ventrolateral prefrontal cortex, while adolescents mainly activated the medial prefrontal cortex during social exclusion (Vijayakumar et al., 2017). Third, while the fixed order for rating valence, arousal, and inclusion avoided confusion during the task, this manipulation introduced a potential order contamination to the image ratings (Kawai et al., 2021; Kurdi et al., 2017). Fourth, our rating task lasted approximately 40 min, which introduced a slight habituation (or fatigue) effect on the image ratings (Table S5 and Fig. S1). Fortunately, images were presented in a random order (i.e., the order differed between participants), which counterbalanced the habituation effect across various images.

Despite these considerations, possible applications of ISIEA are broad. First, it can be applied to studies investigating social and emotional processing in the context of social exclusion/inclusion. These studies include attention allocation, memory, social reward/punishment anticipation and evaluation, and emotion regulation of socially evoked feelings. Second, besides investigating social exclusion/inclusion *per se*, these context-based images can be utilized as affective priming stimuli to induce socially positive or negative emotions, thus facilitating an investigation of follow-up cognitive processes (e.g., decision-making and learning), social behaviors (e.g., prosocial and vengeance trends), and individual characteristics (e.g., self-esteem and state anxiety) influenced by social emotions. Third, the application of the database in clinical research will be beneficial for examining social dysfunctions in clinical populations, such as autism spectrum disorder and alexithymia (Happé et al., 2017).

## Conclusions

The ISIEA database we developed consists of 164 photographs, divided into three social context categories (social exclusion, social neutral, and social inclusion), and is available for free download from 10.5281/zenodo.5496421. By referring to the standardized ratings provided, researchers can choose the appropriate images and flexibly conduct experimental control. We hope that ISIEA will facilitate standardization and comparability across related studies as a valid resource and contribute to promoting the development of socio-emotional studies.

## Supplementary Information

ESM 1 (DOCX 527 kb)

ESM 2 (PDF 509 kb)
