# Supplementary material for: ISIEA: An image database of social inclusion and exclusion in young Asian adults
Source: Behav Res Methods. 2021 Dec 16;54(5):2409–21. doi: 10.3758/s13428-021-01736-w (PMC9579065; doi:10.3758/s13428-021-01736-w)
Supplement: Supplementary file 1 — (DOCX 527 kb) [file 13428_2021_1736_MOESM1_ESM.docx]

ISIEA: An image database of social inclusion and exclusion in young Asian adults

Zixin Zheng, Sijin Li, Licheng Mo, Weimao Chen, Dandan Zhang

Supplementary Material

**Supplementary results**

Table S1. Kolmogorov–Smirnov test for image ratings

| Rating | Exclusion (N = 60) | |  | Neutral (N = 53) | |  | Inclusion (N = 51) | |
| --- | --- | --- | --- | --- | --- | --- | --- | --- |
|  | D | *p* |  | D | *p* |  | D | *p* |
| Valance | 0.065 | 0.959 |  | 0.098 | 0.683 |  | 0.102 | 0.667 |
| Arousal | 0.063 | 0.973 |  | 0.106 | 0.585 |  | 0.134 | 0.320 |
| Inclusion score | 0.058 | 0.987 |  | 0.105 | 0.608 |  | 0.118 | 0.479 |
| Vicarious feeling | 0.071 | 0.922 |  | 0.108 | 0.567 |  | 0.094 | 0.757 |

Table S2. Regression for arousal with (a) valence, (b) inclusion score, and (c) vicarious feeling as predictors (N = 163)

| Dependent variable: arousal | *β* | *β*_standardized_ | t | *p* |
| --- | --- | --- | --- | --- |
| (a) R^2^ = .974, *F*(1,161) = 3024, *p* < .001 |  |  |  |  |
| Valence | -2.491 | -4.269 | -25.418 | **0.000^***^** |
| Valence^2^ | 0.284 | 5.064 | 30.153 | **0.000^***^** |
| (b) R^2^ = .961, *F*(1,161) = 1973, *p* < .001 |  |  |  |  |
| Inclusion score | -2.096 | -4.020 | -25.897 | **0.000^***^** |
| Inclusion scores^2^ | 0.236 | 4.798 | 30.906 | **0.000^***^** |
| (c) R^2^ = .967, *F*(1,162) = 4787, *p* < .001 |  |  |  |  |
| Vicarious feeling | -2.347 | -4.127 | -22.978 | **0.000^***^** |
| Vicarious feeling^2^ | 0.274 | 4.916 | 27.371 | **0.000^***^** |

**p* < 0.05, ***p* < 0.01, and *** *p* < 0.001.

Table S3. Pearson correlations between questionnaire scores and image ratings

| Rating | IRI | |  | LSAS | |
| --- | --- | --- | --- | --- | --- |
|  | *r* | *p*_cor_ |  | *r* | *p*_cor_ |
| **Valence** |  |  |  |  |  |
| Exclusion | -0.255 | **0.005^**^** |  | -0.158 | 0.098 |
| Neutral | 0.078 | 0.408 |  | -0.327 | **0.000^***^** |
| Inclusion | 0.314 | **0.000^***^** |  | -0.062 | 0.518 |
| **Arousal** |  |  |  |  |  |
| Exclusion | 0.052 | 0.572 |  | 0.102 | 0.297 |
| Neutral | -0.100 | 0.297 |  | -0.159 | 0.098 |
| Inclusion | 0.135 | 0.170 |  | 0.083 | 0.396 |
| **Inclusion score** |  |  |  |  |  |
| Exclusion | -0.225 | **0.017^*^** |  | -0.129 | 0.186 |
| Neutral | 0.159 | 0.098 |  | -0.193 | **0.043^*^** |
| Inclusion | 0.341 | **0.000^***^** |  | 0.022 | 0.828 |
| **Vicarious feeling** |  |  |  |  |  |
| Exclusion | -0.269 | **0.003^**^** |  | -0.199 | **0.039^*^** |
| Neutral | 0.006 | 0.937 |  | -0.324 | **0.000^***^** |
| Inclusion | 0.287 | **0.002^**^** |  | 0.101 | 0.297 |

Multiple corrected using FDR method. **p* < 0.05; ***p* < 0.01***; *p* < 0.001

LSAS, the Liebowitz Social Anxiety Scale; IRI, the Interpersonal Reactivity Index.

**Gender effect**

The gender effect was examined for the four ratings utilizing two-way mixed designed ANOVAs. Descriptive data are shown in Table S4. For the valence rating, the main effect of gender was not significant, *F*(1,148) = .07, *p* = .793, $\eta_{p}^{2}$< .001. The interaction between gender and context was not significant, *F*(2,296) = .7, *p* = .440, $\eta_{p}^{2}$= .005. For the arousal rating, the main effect of gender was not significant, *F*(1,148) = .047, *p* = .829, $\eta_{p}^{2}$< .001. The interaction between gender and context was not significant, *F*(2,296) = .781, *p* = .447, $\eta_{p}^{2}$= .005. For the inclusion score, neither the main effect of gender, *F*(1,148) = .596, *p* = .441, $\eta_{p}^{2}$= .004, nor the interaction between gender and context was significant, *F*(2,296) = .447, *p* = .577, $\eta_{p}^{2}$= .003. No significant main effect of gender was found for vicarious feelings, *F*(1,148) = .078, *p* = .780, $\eta_{p}^{2}$ < .001. The interaction between gender and context was also not significant, *F*(2,296) = .313, *p* = .619, $\eta_{p}^{2}$= .002. These results suggested that gender has no effect on the images’ ratings.

Table S4. Descriptive statistics of image ratings by men and women.

| Rating | Men (n = 79) | | |  | Women (n = 71) | | |
| --- | --- | --- | --- | --- | --- | --- | --- |
|  | Exclusion | Neutral | Inclusion |  | Exclusion | Neutral | Inclusion |
| Valance | 3.399 ± 0.936 | 5.153 ± 0.475 | 7.026 ± 0.694 |  | 3.460 ± 0.672 | 5.058 ± 0.353 | 7.105 ± 0.725 |
| Arousal | 4.652 ± 1.243 | 4.200 ± 1.234 | 6.187 ± 1.229 |  | 4.520 ± 1.166 | 4.251 ± 1.077 | 6.358 ± 1.274 |
| Inclusion score | 3.295 ± 0.987 | 5.336 ± 0.717 | 7.318 ± 0.785 |  | 3.332 ± 0.832 | 5.332 ± 0.825 | 7.479 ± 0.758 |
| Vicarious feeling | 3.180 ± 0.787 | 5.015 ± 0.315 | 6.966 ± 0.519 |  | 3.251 ± 0.713 | 4.980 ± 0.271 | 6.970 ± 0.541 |

**Time effect**

To investigate the time effect on the ratings of the three categories of images, we split the rating task into two halves and calculated image ratings in each half. Two-way (2×3) mix design ANOVAs were performed separately for each rating scale, with the context category as the between-images factor and time (the first and the second half of the task) as the within-image factor. Descriptive data are shown in Table S5 and Figure S1.

For valence ratings, the main effect of time was not significant, *F*(1,161) < .001, *p* = .975, $\eta_{p}^{2}$< .001. The interaction between time and context category was significant, *F*(2,161) = 8.3, *p* < .001, $\eta_{p}^{2}$= .093. Simple effect analysis showed that participants rated the social inclusion images less positively in the second half (7.024 ± 0.206) compare to the first half of the task (7.102 ± 0.231), *F*(1,161) = 9.9, *p* = .003, $\eta_{p}^{2}$= .166, and rated the social exclusion images less negatively in the second period (3.457 ± 0.460) compared to the first half of the task (3.400 ± 0.500), *F*(1,161) = 4.6, *p* = .036, $\eta_{p}^{2}$= .073. No significant difference was found for social neutral images, *F*(1,161) = 1.0, *p* = .329, $\eta_{p}^{2}$= .018

For arousal ratings, the main effect of time was not significant, *F*(1,161) = .2, *p* = .698, $\eta_{p}^{2}$< .001. No significant interaction was found between context and time, *F*(2,161) = 1.1, *p* = .337, $\eta_{p}^{2}$= .013.

For inclusion scores, the main effect of time was not significant, *F*(1,161) = 1.3, *p* = .251, $\eta_{p}^{2}$= .008. No significant interaction was found between context and time, *F*(2,161) = 2.9, *p* = .059, $\eta_{p}^{2}$= .035.

For vicarious feelings, the main effect of time was not significant, *F*(1,161) = 1.0, *p* = .312, $\eta_{p}^{2}$= .006. The interaction between time and context category was significant, *F*(2,161) = 17.6, *p* < .001, $\eta_{p}^{2}$= .179. Simple effect analysis showed that participants rated the social inclusion images less positively in the second half (6.917 ± 0.270) compare to the first half (7.019 ± 0.335), *F*(1,161) = 22.3, *p* < .001, $\eta_{p}^{2}$= .308, and rated the social exclusion images less negatively in the second period (3.259 ± 0.354) compared to the first half of the task (3.169 ± 0.386), *F*(1,161) = 11.2, *p* < .001, $\eta_{p}^{2}$= .160. No significant difference was found for social neutral images, *F*(1,161) = 2.2, *p* = .144, $\eta_{p}^{2}$= .041.

In summary, although ANOVAs showed some time effects between the first and the second halves of the task for valence and vicarious feelings, the differences of mean ratings were actually very small between the first and the second half of the rating task (i.e., all differences < 0.1; Table S5). We think this slight time effect produces very limited influences for the image ratings.

Table S5. Descriptive statistics of the image ratings in two halves of the task

| Rating | Exclusion (n = 60) | |  | Neutral (n = 53) | |  | Inclusion (n = 51) | |
| --- | --- | --- | --- | --- | --- | --- | --- | --- |
|  | First | Second |  | First | Second |  | First | Second |
| Valance | 3.400 ± 0.500 | 3.457 ± 0.460 |  | 5.100 ± 0.320 | 5.119 ± 0.278 |  | 7.102 ± 0.231 | 7.024 ± 0.206 |
| Arousal | 4.591 ± 0.252 | 4.591 ± 0.216 |  | 4.198 ± 0.226 | 4.247 ± 0.188 |  | 6.279 ± 0.283 | 6.254 ± 0.263 |
| Inclusion score | 3.283 ± 0.522 | 3.340 ± 0.512 |  | 5.315 ± 0.308 | 5.356 ± 0.247 |  | 7.412 ± 0.258 | 7.373 ± 0.228 |
| Vicarious feeling | 3.169 ± 0.386 | 3.259 ± 0.354 |  | 5.013 ± 0.294 | 4.984 ± 0.246 |  | 7.016 ± 0.335 | 6.917 ± 0.270 |


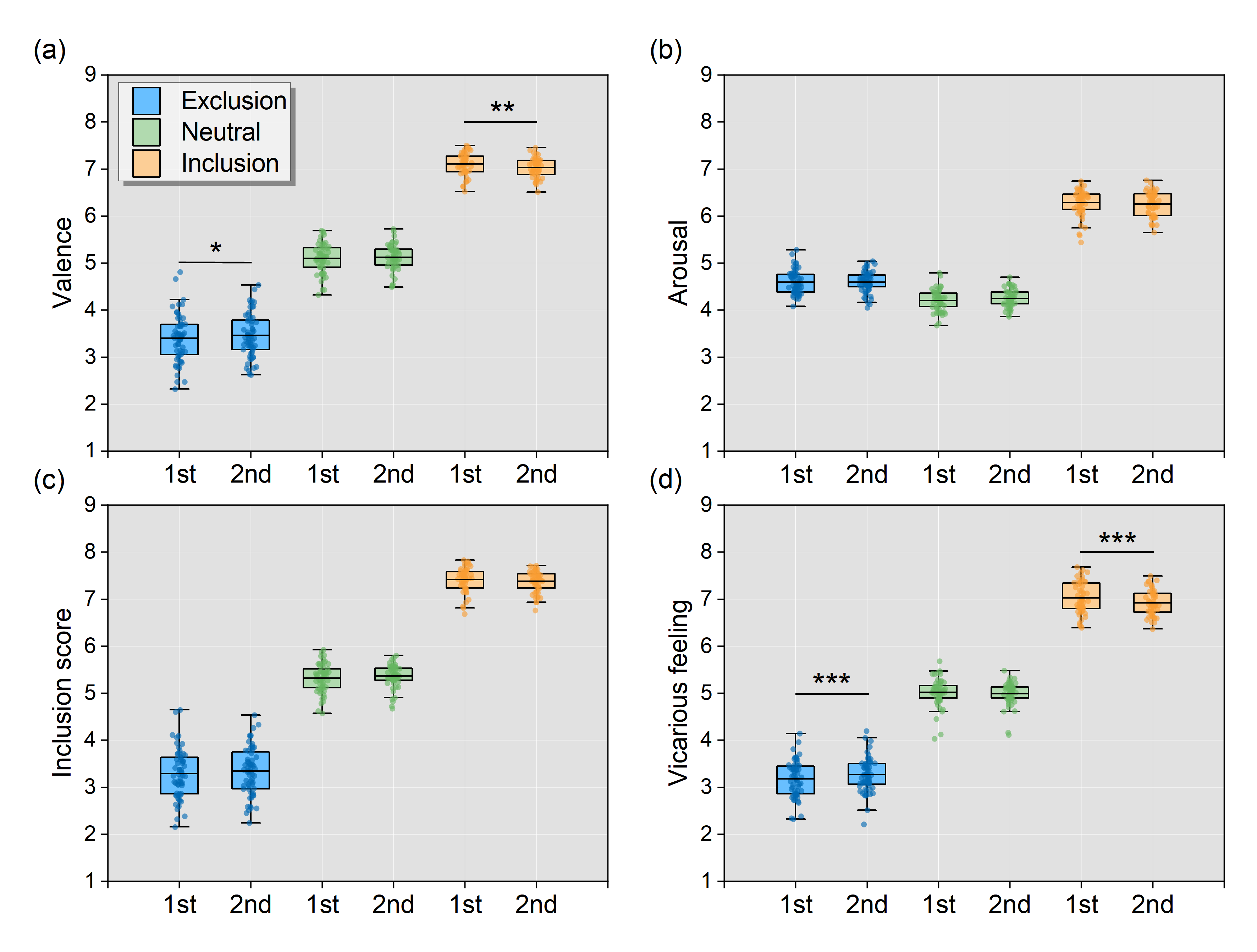


Figure S1. Picture ratings in the first and second half of the rating task for (a) valence, (b) arousal, (c) inclusion, and (d) vicarious feeling. Each dot represents the mean rating of one image, boxes represent the 25th and 75th percentiles, and the whiskers represent upper and lower values within 1.5 inter-quartile range. **p* < 0.05, ***p* < 0.01, and ****p* < 0.001.
