# Supplementary material for: ISIEA: An image database of social inclusion and exclusion in young Asian adults
Source: Behav Res Methods. 2021 Dec 16;54(5):2409–21. doi: 10.3758/s13428-021-01736-w (PMC9579065; doi:10.3758/s13428-021-01736-w)
Supplement: Supplementary file 2 — (PDF 509 kb) [file 13428_2021_1736_MOESM2_ESM.pdf]

Supplementary Material 1: The ratings and physical parameters of ISIEA

ISIEA		Overall (n = 150)								Male (n = 79)								Fmale (n = 71)								Physical parameters						
		Valence		Arousal		Inclusion score		Vicarious feeling		Valence		Arousal		Inclusion score		Vicarious feeling		Valence		Arousal		Inclusion score		Vicarious feeling		Luminance	Contrast	Entropy	JPG_size(bytes)	LAB-L	LAB-A	LAB-B
Category	Picture ID	Mean	SD	Mean	SD	Mean	SD	Mean	SD	Mean	SD	Mean	SD	Mean	SD	Mean	SD	Mean	SD	Mean	SD	Mean	SD	Mean	SD							
Social Exclusion	E1	3.87	1.38	4.76	1.54	3.78	1.55	3.43	1.03	4.00	1.49	4.82	1.51	3.78	1.55	3.48	1.07	3.72	1.24	4.69	1.58	3.77	1.57	3.38	0.99	128.16	81.80	7.28	547690	51.64	1.39	3.96
Social Exclusion	E2	3.31	1.23	4.34	1.67	3.23	1.44	3.13	1.09	3.22	1.32	4.53	1.79	3.13	1.59	3.22	1.01	3.42	1.13	4.13	1.51	3.34	1.25	3.03	1.17	157.84	76.23	7.59	595002	47.37	-0.56	15.63
Social Exclusion	E3	3.31	1.22	4.43	1.66	3.06	1.20	3.41	1.24	3.28	1.21	4.44	1.71	3.08	1.11	3.43	1.25	3.34	1.24	4.41	1.62	3.04	1.30	3.38	1.25	146.13	61.68	7.63	516192	49.31	-1.31	22.86
Social Exclusion	E4	2.83	1.06	4.47	1.82	2.84	1.32	2.85	1.07	2.80	1.07	4.47	1.85	2.87	1.37	2.91	1.03	2.87	1.06	4.48	1.80	2.80	1.26	2.77	1.11	91.02	65.07	7.50	574645	34.64	0.59	7.21
Social Exclusion	E5	3.57	1.25	4.50	1.42	3.59	1.39	3.30	1.15	3.56	1.37	4.57	1.43	3.61	1.52	3.41	1.12	3.58	1.12	4.42	1.42	3.58	1.23	3.18	1.18	108.25	73.79	7.76	564138	56.54	-4.16	15.61
Social Exclusion	E6	3.09	1.17	4.76	1.90	2.83	1.25	2.83	1.07	3.15	1.23	4.81	1.90	2.95	1.35	2.84	1.20	3.03	1.10	4.70	1.91	2.69	1.12	2.83	0.91	111.32	76.66	7.59	682669	32.56	0.51	2.69
Social Exclusion	E7	3.11	1.13	4.42	1.67	2.97	1.35	2.96	1.08	3.22	1.25	4.49	1.73	3.10	1.45	2.89	1.00	2.99	0.98	4.34	1.60	2.83	1.22	3.04	1.16	102.95	58.81	7.64	914086	42.74	-0.98	-1.59
Social Exclusion	E8	3.11	1.39	4.55	1.69	3.13	1.43	2.89	1.06	3.10	1.40	4.42	1.68	3.20	1.58	2.92	1.17	3.11	1.39	4.69	1.70	3.04	1.24	2.85	0.92	135.63	74.50	7.68	681627	41.24	-1.83	11.16
Social Exclusion	E9	2.84	1.18	4.52	1.77	2.68	1.29	2.89	1.13	2.87	1.29	4.46	1.83	2.72	1.34	2.87	1.09	2.80	1.06	4.59	1.71	2.63	1.23	2.90	1.17	126.47	81.62	7.82	621472	56.36	1.82	6.81
Social Exclusion	E10	3.31	1.15	4.55	1.63	3.19	1.22	3.43	1.09	3.35	1.32	4.52	1.72	3.24	1.37	3.51	1.28	3.25	0.92	4.59	1.55	3.13	1.04	3.35	0.83	115.62	76.21	7.90	997803	45.88	0.52	-0.85
Social Exclusion	E11	3.75	1.16	4.66	1.43	3.69	1.42	3.38	1.10	3.72	1.32	4.76	1.51	3.67	1.59	3.39	1.08	3.79	0.96	4.55	1.34	3.72	1.21	3.37	1.12	118.74	64.06	7.80	1297848	47.33	5.52	8.98
Social Exclusion	E12	3.20	1.20	4.71	1.77	2.87	1.22	3.45	1.06	3.11	1.32	4.84	1.75	2.84	1.29	3.44	1.12	3.30	1.06	4.56	1.80	2.90	1.15	3.46	1.00	83.69	54.33	7.40	759600	63.53	0.67	4.43
Social Exclusion	E13	3.15	1.31	4.41	1.70	2.92	1.30	3.04	1.02	3.11	1.45	4.54	1.70	2.94	1.44	3.05	1.12	3.18	1.15	4.27	1.71	2.90	1.12	3.03	0.91	137.20	69.26	7.90	1260367	66.30	-0.99	1.07
Social Exclusion	E14	2.78	1.24	4.68	1.82	2.51	1.09	2.98	1.21	2.80	1.32	4.84	1.78	2.56	1.08	3.08	1.36	2.76	1.15	4.51	1.87	2.46	1.11	2.87	1.01	79.27	57.65	7.56	833269	53.21	3.19	8.72
Social Exclusion	E15	2.92	1.09	4.81	1.87	2.70	1.21	2.85	1.27	2.90	1.23	4.86	1.97	2.70	1.30	2.90	1.46	2.94	0.92	4.76	1.77	2.70	1.10	2.79	1.03	104.98	79.16	7.57	602166	44.36	12.52	32.95
Social Exclusion	E16	3.95	1.31	4.46	1.49	3.82	1.41	3.57	1.22	3.89	1.47	4.44	1.69	3.76	1.47	3.61	1.36	4.01	1.12	4.48	1.24	3.89	1.35	3.52	1.04	98.63	55.98	7.69	1276492	54.86	2.93	13.41
Social Exclusion	E17	4.44	1.79	4.94	1.75	4.35	2.01	3.33	1.18	4.51	1.80	4.97	1.82	4.42	2.05	3.44	1.19	4.37	1.79	4.90	1.68	4.28	1.97	3.21	1.17	138.87	72.88	7.50	807908	39.22	3.05	6.07
Social Exclusion	E18	3.89	1.25	4.27	1.51	3.77	1.44	3.85	1.09	3.70	1.21	4.25	1.64	3.56	1.47	3.80	1.14	4.11	1.26	4.28	1.35	4.01	1.38	3.90	1.03	112.94	82.94	7.40	605388	50.47	5.28	3.45
Social Exclusion	E19	3.96	1.75	4.79	1.68	3.73	1.77	3.12	1.14	4.01	1.71	4.81	1.62	3.78	1.73	3.19	1.13	3.90	1.81	4.77	1.75	3.66	1.83	3.04	1.14	115.47	57.69	7.61	620431	38.08	15.12	20.90
Social Exclusion	E20	3.00	1.11	4.79	1.69	2.82	1.33	2.96	1.12	2.95	1.15	4.81	1.69	2.66	1.30	3.00	1.09	3.06	1.07	4.76	1.71	3.00	1.35	2.92	1.17	163.64	63.69	7.12	535761	42.49	9.33	7.73
Social Exclusion	E21	3.37	1.14	4.59	1.66	3.26	1.36	3.15	1.07	3.42	1.22	4.61	1.72	3.30	1.51	3.06	0.94	3.32	1.07	4.58	1.61	3.21	1.18	3.24	1.20	130.41	69.95	7.66	677814	45.10	4.57	12.39
Social Exclusion	E22	2.66	1.22	4.87	2.04	2.45	1.34	2.27	1.08	2.68	1.29	4.90	2.20	2.47	1.48	2.29	1.13	2.63	1.15	4.85	1.86	2.42	1.18	2.24	1.02	104.27	47.02	7.41	703148	49.99	0.30	15.58
Social Exclusion	E23	3.37	1.29	4.61	1.68	3.26	1.44	3.11	1.16	3.44	1.47	4.71	1.71	3.34	1.48	3.15	1.12	3.28	1.06	4.49	1.65	3.17	1.39	3.07	1.21	136.35	83.55	7.50	680632	59.62	0.35	3.29
Social Exclusion	E24	3.28	1.21	4.51	1.71	3.36	1.35	3.17	1.31	3.24	1.26	4.39	1.87	3.35	1.36	3.01	1.03	3.32	1.14	4.65	1.51	3.37	1.34	3.34	1.55	95.43	64.06	7.74	909657	38.81	11.52	10.41
Social Exclusion	E25	4.65	1.81	5.11	1.59	4.47	1.85	3.56	1.18	4.72	1.84	5.01	1.67	4.52	1.75	3.62	1.25	4.58	1.79	5.23	1.50	4.41	1.96	3.49	1.11	122.96	56.23	7.38	713580	41.66	2.49	8.28
Social Exclusion	E26	3.47	1.36	4.62	1.68	3.22	1.42	3.05	1.18	3.46	1.36	4.48	1.78	3.33	1.56	3.06	1.22	3.49	1.37	4.77	1.56	3.10	1.26	3.03	1.15	90.90	55.63	7.42	839313	63.41	-1.05	8.25
Social Exclusion	E27	3.27	1.14	4.56	1.67	3.21	1.31	3.03	1.10	3.28	1.23	4.59	1.77	3.18	1.24	3.05	1.12	3.27	1.03	4.52	1.56	3.25	1.39	3.00	1.08	104.17	70.45	7.73	773683	52.09	-1.27	6.41
Social Exclusion	E28	3.99	1.45	4.58	1.68	3.76	1.49	3.57	1.16	4.22	1.57	4.68	1.68	3.94	1.53	3.66	1.24	3.75	1.26	4.46	1.69	3.56	1.43	3.46	1.05	108.41	57.88	7.63	811835	58.47	18.17	11.36
Social Exclusion	E29	3.61	1.51	4.73	1.68	3.37	1.48	2.95	1.10	3.76	1.60	4.84	1.66	3.46	1.50	2.84	1.02	3.44	1.38	4.61	1.70	3.27	1.45	3.08	1.18	120.28	56.92	7.77	1062595	50.16	0.41	-15.34
Social Exclusion	E30	3.35	1.36	4.85	1.75	3.27	1.43	2.85	1.36	3.46	1.37	4.65	1.75	3.47	1.46	2.91	1.42	3.23	1.34	5.07	1.74	3.04	1.37	2.77	1.29	93.49	48.85	7.51	818767	37.16	1.09	11.64
Social Exclusion	E31	3.07	1.21	4.60	1.76	2.85	1.25	2.95	1.02	3.10	1.31	4.54	1.80	2.94	1.42	2.97	1.06	3.03	1.11	4.66	1.72	2.75	1.02	2.93	0.98	101.87	65.70	7.80	691078	60.11	-1.36	2.43
Social Exclusion	E32	3.47	1.36	4.43	1.66	3.36	1.41	3.27	1.06	3.52	1.41	4.56	1.71	3.38	1.43	3.30	1.09	3.42	1.30	4.28	1.61	3.34	1.39	3.24	1.04	155.48	61.73	7.46	608655	50.09	-2.91	0.59
Social Exclusion	E33	3.99	1.41	4.70	1.53	3.93	1.57	3.43	1.17	4.06	1.48	4.76	1.52	4.05	1.66	3.54	1.16	3.90	1.33	4.63	1.55	3.80	1.47	3.31	1.17	127.37	72.95	7.77	772201	37.84	-3.80	14.26
Social Exclusion	E34	3.35	1.17	4.77	1.66	3.29	1.31	3.33	1.16	3.33	1.25	4.78	1.63	3.28	1.42	3.24	1.11	3.38	1.09	4.75	1.70	3.31	1.19	3.44	1.22	141.69	60.50	7.54	862276	37.20	2.24	3.44
Social Exclusion	E35	3.41	1.19	4.54	1.60	3.43	1.38	3.27	1.24	3.51	1.26	4.49	1.70	3.49	1.41	3.22	1.07	3.31	1.12	4.59	1.50	3.37	1.34	3.32	1.40	119.54	74.99	7.29	707952	58.03	-4.16	7.74
Social Exclusion	E36	3.52	1.13	4.67	1.54	3.50	1.30	3.52	1.11	3.58	1.30	4.71	1.58	3.46	1.37	3.57	1.16	3.45	0.91	4.62	1.50	3.55	1.24	3.46	1.05	88.87	53.09	7.62	1264796	39.21	-2.24	7.77
Social Exclusion	E37	2.70	1.13	4.92	1.92	2.54	1.31	2.80	1.24	2.75	1.15	4.97	1.90	2.62	1.44	2.72	1.24	2.65	1.12	4.86	1.95	2.45	1.14	2.89	1.24	146.84	60.98	7.71	789613	39.91	-0.97	6.30
Social Exclusion	E38	3.75	1.31	4.64	1.53	3.69	1.37	3.43	1.13	3.73	1.31	4.63	1.60	3.62	1.34	3.30	1.10	3.77	1.32	4.65	1.46	3.77	1.41	3.56	1.16	121.77	72.02	7.59	1142618	37.16	1.15	2.88
Social Exclusion	E39	3.07	1.13	4.61	1.76	3.03	1.25	2.89	1.08	3.10	1.27	4.65	1.79	3.11	1.34	2.81	1.17	3.03	0.97	4.56	1.74	2.94	1.15	2.97	0.99	90.12	54.26	7.51	1151201	48.06	-1.57	-1.95
Social Exclusion	E40	4.03	1.25	4.50	1.59	4.09	1.47	3.69	1.12	4.09	1.39	4.42	1.75	4.11	1.58	3.71	1.16	3.97	1.08	4.59	1.39	4.07	1.35	3.68	1.09	141.58	69.78	7.80	1072626	33.08	-0.20	3.54
Social Exclusion	E41	3.63	1.20	4.37	1.52	3.61	1.27	3.35	1.11	3.63	1.28	4.34	1.53	3.73	1.39	3.32	1.10	3.62	1.11	4.41	1.52	3.48	1.11	3.38	1.11	94.40	58.54	7.71	1195708	36.70	0.63	6.05
Social Exclusion	E42	3.73	1.34	4.59	1.62	3.62	1.55	3.22	1.11	3.72	1.43	4.67	1.72	3.70	1.61	3.23	1.14	3.75	1.25	4.49	1.52	3.54	1.49	3.21	1.08	97.19	66.93	7.76	1121441	37.85	-4.69	5.34
Social Exclusion	E43	3.06	1.44	4.80	1.68	2.88	1.35	3.00	1.25	3.04	1.45	4.95	1.75	2.95	1.46	3.10	1.25	3.08	1.43	4.63	1.59	2.80	1.23	2.89	1.26	91.15	64.11	7.51	653632	42.94	-2.32	6.50
Social Exclusion	E44	3.26	1.14	4.45	1.58	3.11	1.24	3.08	1.05	3.19	1.22	4.47	1.69	3.09	1.35	2.97	1.10	3.34	1.04	4.42	1.45	3.13	1.12	3.20	1.00	115.95	57.97	7.82	986824	47.76	3.15	4.35
Social Exclusion	E45	3.31	1.32	4.28	1.66	3.29	1.30	3.20	1.05	3.32	1.50	4.49	1.73	3.34	1.41	3.16	1.08	3.30	1.10	4.04	1.56	3.23	1.16	3.24	1.02	80.95	61.95	7.47	1067042	44.07	3.76	2.81
Social Exclusion	E46	3.59	1.26	4.25	1.53	3.65	1.52	3.50	1.17	3.54	1.32	4.41	1.52	3.62	1.53	3.59	1.27	3.65	1.20	4.07	1.52	3.68	1.51	3.39	1.06	89.58	69.10	7.74	1260997	59.14	-0.10	4.88
Social Exclusion	E47	3.60	1.12	4.38	1.49	3.59	1.42	3.56	1.14	3.61	1.15	4.44	1.52	3.61	1.52	3.47	1.16	3.59	1.09	4.31	1.47	3.56	1.31	3.66	1.12	91.76	74.15	7.32	1131722	46.72	-0.75	4.20
Social Exclusion	E48	4.01	1.33	4.10	1.53	4.16	1.64	3.83	1.30	3.91	1.45	4.15	1.66	3.99	1.66	3.72	1.26	4.13	1.17	4.04	1.38	4.35	1.60	3.96	1.34	104.01	65.67	7.44	857221	52.75	-3.16	1.77
Social Exclusion	E49	2.55	1.25	4.79	1.88	2.47	1.27	2.45	1.04	2.48	1.34	5.00	1.79	2.48	1.40	2.47	1.00	2.62	1.14	4.56	1.97	2.45	1.11	2.42	1.09	117.24	71.79	7.82	714333	32.02	3.26	4.36
Social Exclusion	E50	2.85	1.15	4.60	1.91	2.66	1.33	2.79	1.14	2.76	1.26	4.65	1.99	2.65	1.46	2.76	1.25	2.96	1.01	4.55	1.83	2.68	1.18	2.82	1.00	145.98	75.83	7.55	561445	39.19	-2.16	2.73
Social Exclusion	E51	3.45	1.19	4.43	1.61	3.31	1.37	3.57	1.18	3.52	1.19	4.30	1.63	3.32	1.46	3.47	1.08	3.37	1.19	4.58	1.58	3.30	1.28	3.69	1.27	114.66	72.73	7.65	538905	45.08	3.25	8.25
Social Exclusion	E52	2.49	1.15	4.81	1.99	2.20	1.17	2.61	1.02	2.53	1.16	4.84	1.96	2.13	1.09	2.61	1.04	2.44	1.13	4.79	2.03	2.28	1.26	2.62	1.01	127.59	62.50	7.84	708541	56.97	-3.33	-4.07
Social Exclusion	E53	3.63	1.24	4.76	1.75	3.43	1.46	3.13	1.13	3.65	1.30	4.70	1.79	3.44	1.47	3.15	1.17	3.61	1.18	4.83	1.72	3.42	1.46	3.10	1.10	79.79	75.53	6.61	632327	62.32	-2.06	-11.20
Social Exclusion	E54	2.88	1.12	4.67	1.80	2.83	1.39	3.04	1.23	2.84	1.07	4.65	1.81	2.84	1.28	3.05	1.27	2.93	1.18	4.69	1.80	2.82	1.52	3.03	1.20	94.57	58.00	7.55	1059579	55.08	-0.88	4.26
Social Exclusion	E55	3.43	1.21	4.69	1.72	3.19	1.41	3.28	1.08	3.43	1.36	4.62	1.85	3.27	1.46	3.27	1.09	3.42	1.04	4.76	1.58	3.10	1.36	3.30	1.07	111.63	79.16	7.49	611772	44.06	-2.01	10.47
Social Exclusion	E56	4.08	1.29	4.47	1.42	3.91	1.41	3.55	1.15	4.14	1.47	4.48	1.55	3.96	1.48	3.67	1.07	4.01	1.08	4.46	1.26	3.85	1.33	3.42	1.23	138.91	70.43	7.32	868069	45.34	6.19	15.96
Social Exclusion	E57	3.51	1.21	4.43	1.67	3.37	1.46	3.29	1.08	3.46	1.27	4.59	1.73	3.37	1.50	3.25	1.14	3.56	1.14	4.25	1.60	3.38	1.42	3.34	1.01	153.25	73.22	7.34	670705	52.64	0.28	8.89
Social Exclusion	E58	4.09	1.20	4.30	1.55	4.00	1.38	4.07	1.12	4.01	1.26	4.30	1.56	3.89	1.30	4.11	1.24	4.18	1.14	4.30	1.55	4.13	1.46	4.03	0.99	133.78	52.38	7.38	594273	42.34	1.43	5.90
Social Exclusion	E59	3.45	1.16	4.39	1.47	3.51	1.50	3.33	1.10	3.37	1.16	4.39	1.57	3.39	1.41	3.35	1.12	3.54	1.16	4.39	1.36	3.63	1.59	3.30	1.09	106.86	69.63	7.80	1178498	54.88	1.63	0.70
Social Exclusion	E60	4.33	1.41	4.59	1.39	4.13	1.55	4.05	1.19	4.28	1.42	4.51	1.36	4.00	1.54	3.91	1.15	4.39	1.40	4.69	1.42	4.28	1.55	4.20	1.23	128.36	64.38	7.87	681756	51.04	2.46	3.73
Social Inclusion	I1	7.33	1.09	6.39	1.65	7.72	1.02	6.91	0.93	7.25	1.06	6.33	1.65	7.61	1.07	6.82	0.90	7.42	1.13	6.46	1.65	7.85	0.97	7.00	0.96	129.47	91.48	7.01	600855	51.90	1.34	6.42
Social Inclusion	I2	7.33	1.02	6.47	1.65	7.62	1.13	7.28	0.89	7.20	1.03	6.34	1.54	7.44	1.24	7.24	0.84	7.46	1.00	6.62	1.77	7.82	0.98	7.32	0.95	148.30	83.99	7.32	774638	49.25	-0.21	11.39
Social Inclusion	I3	7.10	1.16	6.33	1.70	7.49	1.04	7.32	0.94	7.06	1.10	6.25	1.70	7.33	1.05	7.22	0.93	7.14	1.22	6.42	1.71	7.68	1.00	7.44	0.94	102.23	69.91	7.52	712265	35.35	1.19	8.87
Social Inclusion	I4	7.02	1.21	5.97	1.67	7.31	1.18	6.75	0.88	6.95	1.17	5.94	1.66	7.24	1.10	6.72	0.91	7.10	1.27	6.00	1.69	7.39	1.27	6.77	0.87	149.96	85.77	7.20	511135	42.12	4.43	3.35
Social Inclusion	I5	7.23	1.03	6.37	1.61	7.61	1.06	7.10	0.82	7.18	0.98	6.34	1.55	7.54	0.98	7.10	0.78	7.30	1.07	6.39	1.68	7.69	1.14	7.10	0.86	116.62	84.81	7.60	600646	36.27	5.34	7.89
Social Inclusion	I6	6.73	1.19	5.87	1.45	7.01	1.23	6.71	0.90	6.66	1.21	5.92	1.37	6.90	1.27	6.75	0.82	6.82	1.16	5.82	1.54	7.13	1.19	6.68	0.98	85.14	69.66	7.30	625199	60.82	-0.52	2.11
Social Inclusion	I7	7.26	1.20	6.45	1.67	7.65	1.23	7.00	0.96	7.18	1.25	6.32	1.71	7.56	1.28	7.00	1.01	7.35	1.14	6.59	1.63	7.76	1.18	7.00	0.89	79.66	62.52	7.55	886494	48.80	3.06	11.41
Social Inclusion	I8	7.14	1.00	6.53	1.61	7.60	1.11	7.11	0.98	7.15	1.00	6.52	1.52	7.49	1.14	7.06	0.91	7.13	1.01	6.54	1.72	7.72	1.06	7.15	1.05	75.59	48.64	7.45	939285	49.15	10.55	23.23
Social Inclusion	I9	6.99	1.14	6.02	1.75	7.35	1.19	6.71	0.89	6.92	1.12	5.96	1.76	7.20	1.14	6.68	0.87	7.06	1.17	6.08	1.75	7.51	1.24	6.73	0.91	141.74	88.00	7.19	510476	41.35	3.83	11.75
Social Inclusion	I10	6.74	1.03	6.10	1.60	7.04	1.12	6.70	0.90	6.73	0.97	6.00	1.77	6.94	1.14	6.81	0.89	6.75	1.09	6.21	1.40	7.15	1.09	6.58	0.91	121.61	83.69	7.69	747744	43.15	1.90	4.83
Social Inclusion	I11	7.26	1.00	6.44	1.66	7.50	1.04	7.09	0.89	7.22	0.90	6.43	1.67	7.38	0.98	7.11	0.80	7.31	1.10	6.45	1.65	7.63	1.09	7.07	0.99	87.38	80.27	7.47	932652	24.16	8.74	7.02
Social Inclusion	I12	7.11	1.11	6.34	1.72	7.40	1.18	7.21	0.97	6.99	1.10	6.27	1.69	7.32	1.27	7.20	0.95	7.25	1.11	6.42	1.75	7.49	1.07	7.23	1.00	103.47	79.93	7.58	777026	59.77	-0.40	5.48
Social Inclusion	I13	7.15	1.01	6.44	1.64	7.53	1.07	6.93	0.85	7.06	0.95	6.37	1.55	7.39	1.11	6.86	0.83	7.24	1.08	6.52	1.74	7.68	1.01	7.01	0.87	88.32	57.24	7.62	734054	29.86	8.58	11.84
Social Inclusion	I14	7.23	0.98	6.53	1.58	7.57	1.09	7.54	0.90	7.22	0.92	6.51	1.45	7.42	1.21	7.49	0.96	7.24	1.05	6.55	1.72	7.75	0.92	7.59	0.82	151.12	83.02	7.51	668173	54.37	10.71	10.55
Social Inclusion	I15	6.87	1.17	6.11	1.45	7.23	1.16	6.53	0.82	6.67	1.16	5.97	1.37	7.00	1.16	6.56	0.80	7.08	1.16	6.27	1.52	7.49	1.11	6.49	0.84	119.06	68.88	7.80	656933	40.50	-0.73	14.63
Social Inclusion	I16	7.03	1.11	6.37	1.53	7.25	1.24	6.91	1.03	6.96	1.10	6.43	1.57	7.08	1.29	6.90	1.11	7.11	1.12	6.30	1.50	7.45	1.17	6.92	0.94	117.74	51.02	7.60	755675	61.36	-3.85	-1.17
Social Inclusion	I17	6.83	1.17	6.19	1.59	7.09	1.26	6.89	0.92	6.80	1.23	6.10	1.54	7.06	1.27	6.80	0.84	6.86	1.11	6.28	1.66	7.11	1.26	6.99	1.01	100.50	63.70	7.79	816300	53.23	2.09	7.07
Social Inclusion	I18	6.82	1.17	6.09	1.63	7.11	1.20	6.89	0.86	6.68	1.19	5.97	1.69	6.99	1.14	6.84	0.81	6.97	1.13	6.23	1.56	7.25	1.26	6.94	0.92	106.02	79.22	7.69	882124	49.39	2.72	11.23
Social Inclusion	I19	7.18	1.14	6.35	1.64	7.53	1.22	6.95	0.90	7.09	1.16	6.18	1.62	7.41	1.25	6.92	0.87	7.28	1.11	6.55	1.65	7.68	1.19	6.99	0.93	59.69	50.89	7.08	885650	58.42	15.70	11.32
Social Inclusion	I20	7.20	1.08	6.42	1.53	7.61	1.06	6.81	0.94	7.18	0.98	6.37	1.43	7.61	0.99	6.73	0.94	7.23	1.19	6.48	1.65	7.61	1.13	6.90	0.94	73.19	65.07	7.51	781706	45.93	5.25	9.86
Social Inclusion	I21	6.93	1.07	6.17	1.69	7.41	1.02	6.68	0.84	6.86	1.05	6.08	1.69	7.23	1.03	6.59	0.78	7.01	1.09	6.27	1.71	7.61	0.99	6.77	0.90	133.46	69.54	7.89	1146130	45.11	-1.72	2.42
Social Inclusion	I22	7.13	1.02	6.39	1.58	7.43	1.16	6.93	0.83	7.10	1.00	6.44	1.59	7.33	1.16	6.87	0.82	7.15	1.05	6.32	1.58	7.54	1.16	7.00	0.85	98.12	67.29	7.62	1040780	37.76	-0.19	-0.18
Social Inclusion	I23	7.14	1.09	6.41	1.73	7.51	1.12	7.45	1.08	7.13	1.09	6.43	1.82	7.38	1.12	7.47	1.04	7.15	1.09	6.38	1.64	7.65	1.11	7.42	1.13	150.35	73.42	7.58	674672	41.86	0.24	6.13
Social Inclusion	I24	7.13	0.98	6.51	1.56	7.55	1.07	7.20	0.94	7.11	0.91	6.53	1.54	7.46	1.07	7.25	0.98	7.14	1.06	6.48	1.58	7.65	1.06	7.14	0.90	131.22	79.12	7.73	729309	30.58	4.06	2.00
Social Inclusion	I25	6.72	1.11	5.87	1.59	6.85	1.24	6.86	0.86	6.72	0.97	5.77	1.59	6.78	1.09	6.85	0.94	6.72	1.24	5.97	1.60	6.92	1.38	6.87	0.77	120.94	74.68	7.70	1183351	28.26	-0.99	4.43
Social Inclusion	I26	7.02	1.10	6.23	1.62	7.46	1.07	6.86	0.90	6.94	1.09	6.04	1.59	7.29	1.08	6.85	0.92	7.11	1.12	6.44	1.65	7.65	1.04	6.87	0.88	141.70	59.74	7.68	881695	35.35	-3.54	10.51
Social Inclusion	I27	7.05	1.16	6.46	1.52	7.44	1.23	6.65	1.02	6.84	1.19	6.35	1.51	7.18	1.34	6.53	1.14	7.28	1.07	6.58	1.54	7.73	1.03	6.79	0.84	110.94	64.52	7.81	1213705	48.04	-0.70	4.27
Social Inclusion	I28	6.97	1.13	6.30	1.66	7.34	1.11	7.14	0.81	6.87	1.08	6.15	1.55	7.16	1.11	7.14	0.78	7.08	1.18	6.46	1.78	7.54	1.08	7.14	0.85	110.35	74.18	7.79	1038756	54.04	0.88	1.94
Social Inclusion	I29	7.11	1.10	6.38	1.69	7.55	1.05	7.33	0.93	7.09	1.05	6.23	1.74	7.34	1.07	7.28	0.97	7.14	1.15	6.55	1.63	7.77	0.99	7.39	0.89	91.01	57.56	7.71	919047	49.20	0.35	-3.25
Social Inclusion	I30	6.99	1.07	6.20	1.63	7.35	1.07	6.83	0.94	6.90	1.11	6.23	1.65	7.18	1.05	6.75	0.97	7.10	1.03	6.17	1.62	7.55	1.07	6.93	0.90	73.95	58.63	7.46	682887	29.23	0.10	7.56
Social Inclusion	I31	7.05	1.05	6.44	1.66	7.31	1.14	7.03	0.90	7.05	1.10	6.28	1.83	7.27	1.15	7.06	0.95	7.04	0.99	6.62	1.45	7.37	1.12	6.99	0.84	68.99	52.91	7.20	1170251	27.00	-2.32	12.82
Social Inclusion	I32	6.97	1.11	6.28	1.60	7.26	1.29	7.37	0.92	6.87	1.08	6.16	1.77	7.18	1.30	7.28	0.93	7.08	1.13	6.41	1.39	7.35	1.29	7.48	0.89	85.21	63.64	7.50	1182325	28.62	-1.41	6.54
Social Inclusion	I33	7.16	1.11	6.57	1.57	7.56	1.12	7.53	0.92	7.18	1.08	6.53	1.60	7.49	1.05	7.51	0.99	7.14	1.15	6.61	1.54	7.63	1.20	7.55	0.84	118.64	82.25	7.18	946240	34.55	-0.69	3.73
Social Inclusion	I34	7.25	1.20	6.53	1.54	7.60	1.08	7.10	0.87	7.23	1.14	6.43	1.65	7.44	1.15	7.05	0.86	7.28	1.27	6.63	1.41	7.77	0.97	7.15	0.87	132.40	67.94	7.50	808004	60.00	1.01	0.87
Social Inclusion	I35	7.14	1.00	6.26	1.62	7.44	1.10	7.23	0.86	7.10	0.98	6.27	1.62	7.33	1.08	7.30	0.82	7.18	1.03	6.25	1.64	7.56	1.11	7.15	0.91	120.99	78.26	7.49	597736	37.05	-0.02	3.80
Social Inclusion	I36	6.99	1.09	6.25	1.48	7.38	1.20	6.83	0.90	7.01	1.03	6.10	1.41	7.32	1.25	6.86	0.87	6.96	1.15	6.42	1.54	7.45	1.14	6.79	0.94	71.41	64.03	7.49	1220086	36.49	-0.05	-1.20
Social Inclusion	I37	7.13	0.96	6.12	1.67	7.41	1.09	6.48	0.79	7.13	0.90	6.22	1.68	7.35	1.08	6.49	0.82	7.13	1.04	6.01	1.67	7.48	1.11	6.46	0.77	64.61	53.06	7.37	1287893	23.94	0.88	-0.85
Social Inclusion	I38	7.34	1.02	6.45	1.63	7.63	1.14	7.09	0.89	7.27	0.97	6.44	1.61	7.53	1.11	7.15	0.91	7.42	1.08	6.46	1.67	7.73	1.17	7.03	0.86	69.54	59.66	7.38	1117671	37.52	2.00	-2.11
Social Inclusion	I39	7.12	1.06	6.18	1.61	7.34	1.30	6.81	0.81	6.99	1.04	5.87	1.74	7.18	1.29	6.72	0.75	7.27	1.07	6.52	1.38	7.52	1.30	6.92	0.86	83.98	60.15	7.66	625816	46.08	0.77	-0.87
Social Inclusion	I40	7.43	1.11	6.56	1.56	7.66	1.26	7.43	0.96	7.49	1.04	6.67	1.51	7.65	1.05	7.34	0.95	7.37	1.19	6.44	1.62	7.68	1.47	7.52	0.97	90.26	63.71	7.59	652449	41.33	-4.92	11.77
Social Inclusion	I41	6.59	1.08	5.72	1.59	6.91	1.24	6.39	0.93	6.47	1.16	5.67	1.51	6.77	1.37	6.39	0.95	6.73	0.97	5.77	1.68	7.06	1.08	6.38	0.90	88.68	64.11	7.70	680321	50.36	1.88	2.02
Social Inclusion	I42	7.44	1.19	6.63	1.65	7.65	1.30	7.47	1.00	7.41	1.34	6.57	1.69	7.68	1.31	7.52	1.08	7.48	1.01	6.70	1.62	7.61	1.29	7.41	0.90	59.07	49.74	6.97	554003	58.17	-3.78	3.67
Social Inclusion	I43	6.71	0.93	5.72	1.63	6.89	1.17	6.65	0.86	6.72	0.97	5.63	1.75	6.97	1.07	6.63	0.92	6.69	0.89	5.82	1.49	6.79	1.28	6.68	0.79	91.63	65.25	7.63	678563	43.92	-0.29	10.96
Social Inclusion	I44	6.69	1.02	5.72	1.54	6.93	1.26	6.54	0.77	6.58	1.00	5.54	1.55	6.92	1.27	6.54	0.84	6.80	1.05	5.92	1.52	6.94	1.26	6.54	0.67	113.76	80.02	7.57	549766	43.77	-3.37	12.84
Social Inclusion	I45	6.91	1.10	5.89	1.58	7.20	1.18	6.77	0.96	6.80	1.13	5.82	1.35	7.09	1.26	6.80	0.90	7.04	1.06	5.96	1.82	7.32	1.08	6.73	1.03	98.96	76.65	7.51	780217	46.89	2.94	1.81
Social Inclusion	I46	7.07	1.28	6.41	1.49	7.44	1.20	6.73	0.99	6.85	1.37	6.33	1.51	7.30	1.23	6.77	0.99	7.32	1.12	6.51	1.46	7.59	1.14	6.69	0.99	124.83	82.13	7.71	591931	31.64	2.20	7.24
Social Inclusion	I47	7.09	1.11	6.25	1.54	7.52	1.05	6.71	0.77	7.11	1.11	6.25	1.54	7.47	1.01	6.73	0.71	7.07	1.13	6.24	1.54	7.58	1.09	6.68	0.84	142.55	76.42	7.23	1070825	49.12	0.96	0.86
Social Inclusion	I48	7.11	1.09	6.19	1.56	7.47	1.08	6.95	0.90	7.05	1.01	6.25	1.52	7.47	1.01	6.94	0.94	7.18	1.18	6.13	1.61	7.46	1.16	6.96	0.85	106.45	65.70	7.78	875296	34.62	1.00	4.54
Social Inclusion	I49	7.25	1.06	6.59	1.44	7.64	1.17	7.27	0.88	7.19	1.00	6.49	1.46	7.57	1.14	7.23	0.85	7.32	1.13	6.69	1.42	7.72	1.21	7.32	0.92	104.66	61.53	7.79	1062026	32.73	0.24	10.01
Social Inclusion	I50	7.33	1.10	6.43	1.60	7.63	1.09	7.23	0.95	7.41	1.08	6.41	1.64	7.57	1.11	7.25	1.03	7.25	1.12	6.45	1.58	7.69	1.08	7.21	0.86	78.20	72.92	7.20	701252	31.45	-0.25	6.24
Social Inclusion	I51	6.75	0.99	5.83	1.60	7.14	1.10	6.51	0.84	6.73	0.98	5.87	1.60	7.14	1.11	6.46	0.75	6.77	1.00	5.77	1.61	7.14	1.10	6.56	0.94	123.14	90.50	7.23	569191	56.87	3.07	2.60
Social Neutral	N1	5.35	0.94	4.53	1.67	5.51	1.07	5.31	0.68	5.47	0.99	4.56	1.83	5.58	1.11	5.41	0.65	5.23	0.87	4.49	1.48	5.44	1.04	5.21	0.70	84.60	66.44	7.59	1079890	34.78	-1.66	4.58
Social Neutral	N2	5.11	0.69	4.12	1.54	5.33	1.03	5.13	0.64	5.11	0.72	4.04	1.61	5.33	0.94	5.16	0.65	5.11	0.67	4.21	1.47	5.32	1.12	5.08	0.63	171.59	69.77	7.17	526969	49.87	0.12	1.06
Social Neutral	N3	4.57	1.03	3.99	1.56	4.77	1.33	4.88	0.79	4.58	1.03	3.94	1.68	4.78	1.32	4.89	0.70	4.55	1.04	4.04	1.42	4.75	1.36	4.87	0.89	104.10	88.39	7.40	590662	37.94	5.73	6.08
Social Neutral	N4	5.27	0.84	4.38	1.54	5.41	0.98	5.13	0.61	5.44	0.89	4.48	1.50	5.46	1.02	5.18	0.59	5.08	0.75	4.27	1.58	5.37	0.95	5.07	0.62	157.47	74.77	7.55	670250	45.88	3.53	-0.18
Social Neutral	N5	5.34	0.93	4.12	1.65	5.56	1.21	5.13	0.55	5.43	1.02	4.08	1.72	5.59	1.24	5.13	0.54	5.24	0.80	4.17	1.59	5.52	1.19	5.13	0.56	112.84	57.57	7.69	752094	61.18	0.16	0.75
Social Neutral	N6	4.92	0.82	4.08	1.50	5.21	1.11	4.89	0.56	4.89	0.92	3.96	1.51	5.14	1.14	4.86	0.64	4.96	0.71	4.21	1.49	5.28	1.09	4.93	0.46	84.85	65.98	7.49	937298	36.98	3.40	6.27
Social Neutral	N7	5.12	0.66	4.01	1.57	5.37	1.05	5.03	0.52	5.19	0.79	3.96	1.69	5.34	1.13	5.01	0.49	5.04	0.46	4.07	1.43	5.39	0.96	5.04	0.55	97.82	77.92	7.49	551623	34.04	5.01	12.93
Social Neutral	N8	5.17	0.94	4.20	1.64	5.47	1.07	4.97	0.67	5.15	1.08	4.09	1.80	5.51	1.07	4.92	0.59	5.18	0.76	4.32	1.44	5.44	1.07	5.03	0.74	143.82	87.27	7.00	530117	43.32	2.54	13.65
Social Neutral	N9	5.35	0.81	4.19	1.54	5.53	1.03	5.22	0.70	5.43	0.94	4.09	1.66	5.53	1.11	5.28	0.80	5.27	0.63	4.31	1.41	5.54	0.95	5.15	0.58	125.86	82.94	7.69	623802	38.01	-0.37	6.39
Social Neutral	N10	4.95	0.96	4.11	1.56	5.13	1.10	4.84	0.91	4.90	1.06	4.00	1.71	5.08	1.07	4.81	0.94	5.01	0.84	4.23	1.37	5.20	1.13	4.87	0.88	122.26	76.59	7.74	654394	47.27	2.57	4.04
Social Neutral	N11	5.13	0.96	3.97	1.54	5.35	1.18	5.01	0.64	5.04	1.13	3.84	1.69	5.24	1.16	5.05	0.62	5.23	0.72	4.11	1.36	5.48	1.19	4.96	0.66	92.83	72.38	7.49	705349	28.33	8.18	12.34
Social Neutral	N12	5.15	0.87	4.13	1.54	5.38	1.01	4.76	0.68	5.19	0.89	4.00	1.70	5.34	0.96	4.84	0.63	5.10	0.85	4.28	1.33	5.42	1.08	4.68	0.73	113.32	82.33	7.63	711352	68.95	0.51	3.01
Social Neutral	N13	4.71	0.90	4.15	1.52	5.22	1.13	4.65	0.83	4.65	1.05	4.05	1.54	5.13	1.14	4.70	0.91	4.79	0.70	4.25	1.49	5.32	1.13	4.61	0.75	151.12	77.57	7.53	637865	26.88	8.88	13.06
Social Neutral	N14	4.93	0.59	4.25	1.40	5.11	0.85	4.93	0.59	4.99	0.57	4.15	1.55	5.11	0.85	4.94	0.65	4.86	0.62	4.35	1.22	5.11	0.85	4.92	0.53	90.86	70.75	7.60	626308	33.61	5.69	10.72
Social Neutral	N15	4.88	0.67	4.31	1.51	5.15	0.90	4.68	0.86	4.91	0.74	4.25	1.62	5.14	0.75	4.71	0.88	4.85	0.58	4.37	1.38	5.17	1.04	4.65	0.85	82.97	65.64	7.44	632334	48.43	4.83	12.59
Social Neutral	N16	5.35	0.95	4.15	1.60	5.50	1.19	5.11	0.77	5.54	0.97	4.15	1.71	5.57	1.15	5.22	0.75	5.14	0.88	4.15	1.48	5.42	1.24	4.99	0.78	105.85	69.78	7.76	778426	53.39	-1.70	4.98
Social Neutral	N17	5.13	0.86	4.37	1.48	5.27	1.06	5.28	0.75	5.27	0.92	4.37	1.64	5.34	1.05	5.28	0.73	4.99	0.77	4.38	1.28	5.18	1.07	5.28	0.78	92.96	70.40	7.68	709809	52.15	-3.09	1.80
Social Neutral	N18	5.13	0.70	4.23	1.58	5.41	1.09	5.00	0.67	5.20	0.81	4.28	1.63	5.38	1.03	4.96	0.67	5.06	0.56	4.17	1.53	5.44	1.16	5.04	0.66	115.40	71.25	7.90	977945	48.56	-0.54	3.15
Social Neutral	N19	5.61	1.04	4.23	1.78	5.71	1.28	5.36	0.88	5.71	1.06	3.99	1.88	5.77	1.25	5.33	0.81	5.49	1.01	4.49	1.63	5.63	1.32	5.39	0.95	69.27	64.81	7.37	732588	38.04	0.22	2.21
Social Neutral	N20	5.68	1.12	4.17	1.87	5.81	1.38	5.30	0.79	5.72	1.18	4.11	2.01	5.85	1.42	5.23	0.72	5.63	1.06	4.24	1.71	5.76	1.35	5.38	0.87	65.58	54.48	7.34	738154	34.63	9.28	8.87
Social Neutral	N21	5.29	1.08	4.03	1.68	5.65	1.25	5.19	0.70	5.43	1.20	4.04	1.71	5.71	1.31	5.24	0.66	5.13	0.93	4.01	1.65	5.58	1.18	5.14	0.74	82.08	59.57	7.51	867698	40.12	4.63	15.61
Social Neutral	N22	5.23	0.94	4.31	1.64	5.58	1.14	4.95	0.62	5.29	1.02	4.32	1.61	5.59	1.21	4.92	0.75	5.17	0.86	4.30	1.68	5.56	1.07	4.97	0.45	119.35	76.53	7.69	817519	51.99	0.19	4.08
Social Neutral	N23	5.41	0.91	4.31	1.64	5.65	1.15	5.21	0.75	5.39	0.90	4.15	1.64	5.46	1.07	5.24	0.82	5.44	0.92	4.49	1.62	5.86	1.20	5.18	0.66	130.34	69.24	7.65	613524	41.77	3.02	1.78
Social Neutral	N24	5.06	0.86	4.08	1.59	5.29	1.05	4.91	0.69	5.11	0.88	3.96	1.76	5.37	1.00	4.92	0.84	5.00	0.85	4.21	1.37	5.21	1.11	4.90	0.45	126.70	67.92	7.60	656641	52.25	-0.44	1.82
Social Neutral	N25	5.31	0.96	4.15	1.53	5.47	1.09	5.03	0.68	5.34	0.95	4.00	1.68	5.39	1.02	5.08	0.84	5.27	0.99	4.31	1.34	5.55	1.17	4.97	0.41	118.36	74.44	7.82	775239	43.03	-1.58	2.34
Social Neutral	N26	4.58	1.01	4.41	1.56	4.80	1.42	5.36	0.81	4.75	1.02	4.42	1.62	4.96	1.33	5.37	0.88	4.39	0.96	4.41	1.50	4.62	1.51	5.35	0.72	92.75	66.70	7.78	635736	36.66	0.02	-0.85
Social Neutral	N27	5.30	0.84	4.49	1.55	5.48	1.10	4.83	0.80	5.46	0.97	4.56	1.72	5.59	1.16	4.84	0.84	5.13	0.63	4.42	1.33	5.35	1.03	4.83	0.76	82.46	46.64	7.17	940559	42.16	-1.53	4.13
Social Neutral	N28	5.15	0.94	4.25	1.59	5.23	1.25	5.13	0.64	5.14	1.08	4.20	1.76	5.27	1.25	5.20	0.63	5.15	0.77	4.30	1.40	5.18	1.26	5.06	0.65	94.98	43.22	7.32	1095117	51.74	-3.21	12.02
Social Neutral	N29	5.15	0.79	4.30	1.55	5.33	1.00	5.36	0.82	5.30	0.93	4.37	1.74	5.29	1.00	5.47	0.90	4.99	0.57	4.23	1.30	5.37	1.00	5.24	0.71	127.41	72.07	7.61	711880	54.57	-1.49	7.42
Social Neutral	N30	5.65	1.10	4.72	1.77	5.75	1.23	5.20	0.79	5.77	1.20	4.81	1.96	5.82	1.28	5.29	0.88	5.51	0.97	4.62	1.53	5.68	1.17	5.10	0.66	127.43	74.11	7.56	1076754	44.84	-9.36	16.51
Social Neutral	N31	5.34	0.92	4.46	1.65	5.47	1.04	5.13	0.67	5.47	0.95	4.42	1.70	5.49	1.09	5.16	0.76	5.20	0.87	4.51	1.60	5.44	1.00	5.08	0.55	104.29	66.21	7.74	1109999	37.83	1.03	6.46
Social Neutral	N32	5.10	0.69	4.15	1.55	5.34	1.02	4.99	0.46	5.13	0.69	4.04	1.65	5.34	0.93	5.01	0.54	5.07	0.70	4.28	1.42	5.34	1.11	4.96	0.36	88.98	61.70	7.65	708654	48.29	0.54	0.52
Social Neutral	N33	5.47	1.04	4.40	1.56	5.71	1.21	5.13	0.61	5.52	1.14	4.29	1.74	5.76	1.29	5.11	0.60	5.41	0.92	4.52	1.34	5.65	1.12	5.15	0.62	104.19	80.65	7.64	1137374	50.66	3.22	-1.87
Social Neutral	N34	4.46	0.99	4.14	1.43	4.81	1.21	4.14	1.01	4.44	1.11	4.14	1.53	4.73	1.23	4.10	1.06	4.48	0.86	4.14	1.32	4.90	1.20	4.18	0.96	125.49	68.82	7.87	1477022	63.52	0.96	3.05
Social Neutral	N35	5.39	0.84	4.27	1.63	5.56	1.11	5.10	0.55	5.41	0.91	4.13	1.76	5.53	1.01	5.11	0.64	5.38	0.74	4.44	1.47	5.59	1.21	5.08	0.44	134.26	79.78	7.56	1058496	42.85	3.97	7.68
Social Neutral	N36	4.95	0.71	4.22	1.42	5.23	1.04	4.98	0.64	4.95	0.82	4.25	1.55	5.16	1.02	4.91	0.75	4.96	0.57	4.18	1.28	5.30	1.06	5.06	0.48	106.76	68.65	7.88	1263949	49.29	0.00	2.35
Social Neutral	N37	5.07	0.70	4.21	1.62	5.37	1.06	5.13	0.81	5.06	0.88	4.10	1.68	5.34	1.10	5.13	0.91	5.07	0.43	4.34	1.54	5.39	1.04	5.14	0.68	90.02	53.53	7.62	927502	43.72	-1.16	11.28
Social Neutral	N38	5.13	0.62	4.16	1.54	5.37	0.95	5.06	0.53	5.19	0.72	4.06	1.66	5.35	0.96	5.05	0.48	5.06	0.48	4.27	1.40	5.39	0.95	5.07	0.59	117.04	59.67	7.73	816070	27.52	-2.09	-0.39
Social Neutral	N39	5.53	0.97	4.65	1.59	5.58	1.02	5.59	0.84	5.67	1.13	4.52	1.77	5.62	1.07	5.65	0.91	5.37	0.74	4.79	1.35	5.54	0.97	5.54	0.75	125.08	79.02	7.68	726297	30.69	-3.76	4.96
Social Neutral	N40	4.91	0.96	4.31	1.63	5.22	1.12	4.87	0.87	5.01	0.97	4.13	1.73	5.23	1.01	4.92	0.80	4.80	0.95	4.52	1.49	5.21	1.23	4.82	0.95	102.63	60.21	7.74	873623	32.40	-0.90	4.42
Social Neutral	N41	5.12	0.71	4.33	1.51	5.40	0.99	5.04	0.52	5.22	0.75	4.41	1.63	5.41	0.91	5.10	0.65	5.01	0.67	4.25	1.37	5.39	1.08	4.97	0.29	120.25	66.79	7.84	759033	45.75	-2.10	8.01
Social Neutral	N42	4.85	0.78	4.31	1.34	5.04	1.09	4.85	0.81	4.87	0.82	4.19	1.48	5.10	1.06	4.82	0.86	4.82	0.72	4.44	1.17	4.97	1.13	4.87	0.76	105.28	62.96	7.80	1284047	31.19	1.66	5.27
Social Neutral	N43	4.64	1.08	4.29	1.59	4.95	1.40	4.77	0.84	4.63	1.11	4.29	1.69	4.81	1.31	4.81	0.96	4.65	1.06	4.28	1.50	5.11	1.48	4.73	0.68	67.54	62.63	7.09	953053	53.94	-1.62	0.86
Social Neutral	N44	4.82	0.69	4.15	1.44	5.08	0.87	4.73	0.60	4.71	0.83	4.39	1.35	5.00	0.83	4.68	0.65	4.94	0.44	3.87	1.50	5.17	0.91	4.77	0.54	74.09	60.15	7.36	1027349	61.18	-1.57	0.04
Social Neutral	N45	4.80	0.85	4.18	1.39	5.01	1.03	4.72	0.73	4.82	0.92	4.09	1.43	5.03	1.05	4.81	0.74	4.77	0.78	4.28	1.34	4.99	1.01	4.62	0.70	78.97	62.10	7.48	1056963	46.54	2.74	7.26
Social Neutral	N46	5.28	0.91	4.24	1.49	5.47	1.11	4.95	0.54	5.28	0.85	4.14	1.63	5.43	1.18	4.99	0.52	5.28	0.97	4.35	1.32	5.51	1.03	4.92	0.55	110.44	70.63	7.83	664563	41.62	1.35	-1.96
Social Neutral	N47	5.51	1.04	4.13	1.73	5.68	1.33	5.11	0.55	5.59	1.09	4.20	1.79	5.70	1.35	5.14	0.42	5.42	0.98	4.06	1.66	5.66	1.32	5.08	0.67	76.22	59.92	7.44	582826	66.23	-3.83	5.92
Social Neutral	N48	4.89	0.67	4.02	1.50	5.21	1.03	4.98	0.50	4.85	0.79	3.97	1.55	5.24	1.04	4.95	0.64	4.94	0.50	4.07	1.46	5.18	1.03	5.01	0.27	131.90	68.86	7.73	530910	40.08	0.04	1.64
Social Neutral	N49	5.05	0.91	3.94	1.56	5.25	1.08	4.87	0.77	5.16	0.98	3.84	1.59	5.39	1.03	4.84	0.84	4.93	0.82	4.06	1.54	5.10	1.12	4.90	0.70	150.76	73.71	7.75	560919	41.03	-2.49	10.08
Social Neutral	N50	5.05	0.81	3.97	1.61	5.28	1.10	4.87	0.67	5.01	0.86	3.71	1.70	5.25	0.99	4.86	0.73	5.08	0.75	4.27	1.47	5.31	1.21	4.89	0.60	101.96	70.72	7.48	578694	34.98	-1.60	10.06
Social Neutral	N51	5.07	0.82	4.10	1.58	5.39	1.21	5.09	0.67	5.10	0.87	3.97	1.63	5.52	1.21	5.09	0.66	5.04	0.76	4.24	1.52	5.25	1.20	5.08	0.67	162.12	64.14	7.43	1029075	39.67	3.48	4.67
Social Neutral	N52	4.92	0.82	4.25	1.49	5.23	1.08	4.98	0.60	4.91	1.02	4.35	1.59	5.18	1.13	4.96	0.72	4.93	0.52	4.14	1.39	5.28	1.03	5.00	0.41	96.77	59.07	7.72	758671	57.80	0.11	-1.08
Social Neutral	N53	4.42	1.14	4.23	1.51	4.65	1.28	4.07	1.14	4.35	1.24	4.43	1.51	4.49	1.29	4.06	1.16	4.49	1.03	4.01	1.50	4.82	1.25	4.07	1.13	99.75	67.69	7.70	897389	50.91	0.97	-0.93

ISIEA		Face (n = 50)				Context (n = 50)					
		Valence		Arousal		Valence		Arousal		Inclusion score	
Category	Picture ID	Mean	SD	Mean	SD	Mean	SD	Mean	SD	Mean	SD
Social Exclusion	E1	4.92	0.99	4.24	1.78	4.12	1.67	4.76	1.59	3.32	1.49
Social Exclusion	E2	4.16	1.02	4.24	1.57	3.52	1.31	5.00	1.67	3.04	1.26
Social Exclusion	E3	4.10	0.97	3.82	1.54	3.64	1.31	4.64	1.43	3.42	1.37
Social Exclusion	E4	3.20	1.07	3.86	1.78	3.60	1.31	4.62	1.64	3.14	1.25
Social Exclusion	E5	3.96	1.20	4.22	1.53	3.96	1.48	4.52	1.52	3.58	1.25
Social Exclusion	E6	3.08	1.18	4.26	1.83	3.08	1.21	4.84	1.75	2.70	1.28
Social Exclusion	E7	3.62	1.11	3.58	1.74	3.54	1.39	4.70	1.67	2.94	1.08
Social Exclusion	E8	3.08	1.01	3.66	1.55	3.26	1.35	5.08	1.50	3.18	1.32
Social Exclusion	E9	3.36	1.21	3.68	1.70	3.10	1.27	5.00	1.73	2.70	1.33
Social Exclusion	E10	3.84	1.00	3.46	1.62	4.10	1.62	4.84	1.74	3.38	1.28
Social Exclusion	E11	4.46	1.05	4.06	1.57	4.36	1.50	4.36	1.69	3.76	1.48
Social Exclusion	E12	4.08	1.10	4.16	1.67	3.16	1.22	5.00	1.58	2.64	1.23
Social Exclusion	E13	3.70	1.02	3.52	1.58	3.38	1.48	4.78	1.71	3.12	1.35
Social Exclusion	E14	3.40	1.14	4.60	1.86	3.16	1.65	5.06	1.81	2.44	1.28
Social Exclusion	E15	3.92	1.18	3.78	1.53	3.34	1.59	5.20	1.75	2.78	1.35
Social Exclusion	E16	3.68	1.10	3.84	1.93	4.46	1.28	4.54	1.71	4.18	1.54
Social Exclusion	E17	4.86	0.95	4.06	1.58	4.80	1.81	5.00	1.60	4.26	1.90
Social Exclusion	E18	3.80	0.99	3.74	1.37	4.26	1.37	4.56	1.68	4.18	1.67
Social Exclusion	E19	4.02	1.17	3.94	1.49	4.22	1.50	4.52	1.63	3.68	1.38
Social Exclusion	E20	3.72	0.97	4.06	1.73	2.98	1.27	4.88	1.67	2.58	1.20
Social Exclusion	E21	4.32	0.87	3.72	1.64	3.56	1.16	4.80	1.60	3.08	1.47
Social Exclusion	E22	3.60	1.18	3.94	1.53	2.50	1.15	5.20	1.92	2.06	1.17
Social Exclusion	E23	3.54	1.01	3.90	1.58	3.84	1.18	4.94	1.68	3.40	1.36
Social Exclusion	E24	3.58	1.18	4.08	1.84	3.70	1.57	4.62	1.62	3.08	1.38
Social Exclusion	E25	3.72	0.99	3.98	1.71	5.60	1.97	5.38	2.00	5.70	2.13
Social Exclusion	E26	3.18	1.19	3.76	1.74	3.98	1.44	4.94	1.63	3.18	1.17
Social Exclusion	E27	3.20	1.31	4.34	1.88	4.16	1.42	4.64	1.51	3.72	1.54
Social Exclusion	E28	5.00	1.03	4.12	1.57	4.10	1.45	4.64	1.74	3.44	1.46
Social Exclusion	E29	3.74	1.24	3.74	1.69	3.36	1.55	5.04	1.95	2.98	1.51
Social Exclusion	E30	3.52	1.52	4.46	2.02	4.32	1.41	4.54	1.57	3.52	1.22
Social Exclusion	E31	3.38	1.03	4.00	1.69	3.32	1.49	4.68	1.85	2.92	1.35
Social Exclusion	E32	4.36	1.05	3.80	1.47	3.70	1.56	4.84	1.62	3.24	1.19
Social Exclusion	E33	3.88	1.30	3.94	1.83	4.12	1.44	4.50	1.50	3.68	1.29
Social Exclusion	E34	4.04	1.01	3.92	1.61	4.06	1.46	4.44	1.63	3.58	1.37
Social Exclusion	E35	3.76	1.22	3.56	1.75	3.94	1.38	4.80	1.60	3.48	1.52
Social Exclusion	E36	3.86	1.26	3.80	1.53	4.46	1.28	4.44	1.61	4.14	1.50
Social Exclusion	E37	4.08	1.23	3.94	1.80	2.64	1.31	4.94	1.92	2.00	1.21
Social Exclusion	E38	3.82	0.92	3.68	1.53	4.40	1.31	4.56	1.40	4.06	1.17
Social Exclusion	E39	2.98	1.25	4.08	1.99	3.56	1.50	4.80	1.74	3.08	1.23
Social Exclusion	E40	3.74	1.24	3.80	1.80	4.14	1.21	4.54	1.61	3.98	1.35
Social Exclusion	E41	3.98	1.32	3.70	1.71	3.92	1.44	4.62	1.76	3.66	1.35
Social Exclusion	E42	3.96	1.05	3.78	1.86	4.02	1.67	4.98	1.70	3.38	1.14
Social Exclusion	E43	3.78	1.17	3.80	1.86	4.82	1.75	4.78	1.28	4.52	1.66
Social Exclusion	E44	3.32	1.08	3.64	1.75	3.78	1.39	4.80	1.58	3.28	1.36
Social Exclusion	E45	3.68	1.38	4.14	1.99	3.56	1.40	4.56	1.76	3.10	1.31
Social Exclusion	E46	3.76	1.22	3.82	1.87	4.16	1.11	4.40	1.40	3.84	1.18
Social Exclusion	E47	4.02	1.04	3.60	1.50	4.58	1.63	4.62	1.59	3.76	1.42
Social Exclusion	E48	4.06	1.15	4.00	1.71	4.32	1.45	4.42	1.72	3.88	1.32
Social Exclusion	E49	2.88	1.22	4.04	2.04	3.22	1.20	4.98	1.61	2.82	1.08
Social Exclusion	E50	3.88	1.32	4.12	1.83	3.38	1.43	4.72	1.81	2.86	1.44
Social Exclusion	E51	4.02	1.10	4.16	1.67	4.06	1.58	4.84	1.54	3.46	1.46
Social Exclusion	E52	3.22	1.15	4.12	1.93	2.60	1.21	4.96	2.01	2.16	1.25
Social Exclusion	E53	3.72	1.25	4.52	1.89	4.06	1.19	4.58	1.16	3.94	1.33
Social Exclusion	E54	4.26	1.12	4.24	1.72	2.94	1.20	4.76	1.92	2.34	1.17
Social Exclusion	E55	4.54	1.20	3.74	1.45	3.24	1.27	5.04	1.78	2.78	1.33
Social Exclusion	E56	4.64	1.03	4.06	1.63	4.14	1.33	4.90	1.52	3.64	1.48
Social Exclusion	E57	3.80	1.50	4.28	2.06	3.66	1.47	4.56	1.66	3.34	1.29
Social Exclusion	E58	4.34	0.98	3.74	1.52	4.56	1.37	4.52	1.43	3.84	1.30
Social Exclusion	E59	3.52	1.15	4.34	1.76	4.38	1.19	4.50	1.63	4.24	1.41
Social Exclusion	E60	5.04	1.03	4.28	1.77	4.24	1.19	4.30	1.42	3.80	1.33
Social Inclusion	I1	6.62	1.24	5.92	1.63	7.36	0.92	6.60	1.60	7.70	0.91
Social Inclusion	I2	6.94	1.04	6.24	1.61	7.18	0.85	6.28	1.54	7.62	0.95
Social Inclusion	I3	7.12	0.98	6.54	1.43	6.82	0.80	6.36	1.43	6.96	1.20
Social Inclusion	I4	6.68	1.00	5.86	1.55	6.68	0.96	5.78	1.48	6.92	1.05
Social Inclusion	I5	7.26	0.90	6.16	1.77	6.76	0.85	5.92	1.61	7.12	0.94
Social Inclusion	I6	6.62	1.16	6.04	1.43	5.88	0.92	5.24	1.59	6.62	1.09
Social Inclusion	I7	6.86	0.83	6.02	1.52	7.00	1.09	6.48	1.46	7.60	1.09
Social Inclusion	I8	6.66	0.82	5.78	1.50	7.14	0.99	6.40	1.55	7.58	1.05
Social Inclusion	I9	6.10	1.04	5.22	1.56	6.98	1.02	6.20	1.70	7.42	1.03
Social Inclusion	I10	6.38	0.97	5.50	1.61	6.78	1.02	6.08	1.61	7.12	1.08
Social Inclusion	I11	7.26	0.85	6.46	1.56	7.00	0.88	6.42	1.53	7.36	1.08
Social Inclusion	I12	7.02	0.94	6.36	1.54	6.76	0.87	6.14	1.73	7.28	1.03
Social Inclusion	I13	6.60	0.95	5.92	1.51	7.04	1.12	6.60	1.57	7.62	0.97
Social Inclusion	I14	7.62	0.86	7.02	1.48	6.82	0.83	5.96	1.48	7.14	0.90
Social Inclusion	I15	5.54	0.97	4.96	1.62	6.94	1.04	6.36	1.50	7.60	0.99
Social Inclusion	I16	6.34	1.06	5.56	1.54	6.56	1.23	5.86	1.60	7.20	0.97
Social Inclusion	I17	6.76	0.92	5.94	1.61	7.16	0.87	6.26	1.70	7.50	1.11
Social Inclusion	I18	6.78	0.91	5.94	1.54	6.28	0.99	5.56	1.55	6.52	1.18
Social Inclusion	I19	6.72	0.97	5.48	1.54	7.18	0.90	6.28	1.67	7.52	0.95
Social Inclusion	I20	6.60	0.83	5.40	1.53	7.34	0.92	6.66	1.62	7.74	0.94
Social Inclusion	I21	6.78	1.00	5.62	1.55	6.84	0.96	6.48	1.40	7.40	0.97
Social Inclusion	I22	7.12	1.00	6.10	1.76	6.86	0.86	6.16	1.50	7.16	1.10
Social Inclusion	I23	7.24	1.06	6.78	1.58	7.02	0.89	6.44	1.57	7.34	1.24
Social Inclusion	I24	7.08	0.80	6.36	1.38	6.64	1.48	6.26	1.47	6.94	1.62
Social Inclusion	I25	6.74	0.60	5.82	1.24	6.74	0.88	6.20	1.33	6.96	1.09
Social Inclusion	I26	7.04	1.03	6.16	1.57	6.98	0.92	6.42	1.36	7.30	1.13
Social Inclusion	I27	6.34	0.80	5.22	1.56	7.12	0.90	6.48	1.46	7.56	0.97
Social Inclusion	I28	7.06	1.04	6.30	1.43	7.04	0.83	6.40	1.40	7.20	1.09
Social Inclusion	I29	6.98	0.98	6.40	1.62	7.08	1.03	6.66	1.42	7.32	1.04
Social Inclusion	I30	7.16	0.84	5.84	1.65	6.86	1.01	6.32	1.52	7.32	0.94
Social Inclusion	I31	6.92	0.94	5.96	1.56	6.96	1.01	6.68	1.54	7.30	1.22
Social Inclusion	I32	7.06	1.17	6.40	1.65	6.86	1.05	6.26	1.77	7.16	1.24
Social Inclusion	I33	7.24	0.94	6.66	1.69	6.84	1.11	6.40	1.58	7.32	1.24
Social Inclusion	I34	7.06	1.00	6.20	1.74	6.86	1.18	6.20	1.40	7.10	1.22
Social Inclusion	I35	7.28	0.86	6.70	1.61	6.88	0.82	6.22	1.71	7.32	0.84
Social Inclusion	I36	6.64	1.06	5.80	1.44	6.80	0.90	6.16	1.39	7.32	0.89
Social Inclusion	I37	6.50	0.91	5.44	1.47	6.90	0.86	5.78	1.79	7.34	1.17
Social Inclusion	I38	6.60	1.14	6.02	1.58	7.14	1.05	6.54	1.40	7.68	1.12
Social Inclusion	I39	6.72	0.93	5.84	1.33	6.82	1.00	6.16	1.25	7.06	1.20
Social Inclusion	I40	7.32	0.98	6.70	1.59	6.04	1.58	5.98	1.22	6.38	1.73
Social Inclusion	I41	6.20	1.14	5.46	1.67	6.30	1.02	5.18	1.56	6.60	1.25
Social Inclusion	I42	6.84	1.41	6.56	1.47	7.16	0.96	6.46	1.71	7.62	1.09
Social Inclusion	I43	6.42	0.70	5.46	1.45	6.00	1.09	5.22	1.46	6.36	1.31
Social Inclusion	I44	6.16	1.28	5.16	1.54	6.08	1.09	5.72	1.31	6.60	1.01
Social Inclusion	I45	6.40	0.83	5.42	1.47	6.26	1.14	5.58	1.43	6.62	1.31
Social Inclusion	I46	6.68	0.94	5.78	1.53	7.04	1.09	6.38	1.43	7.54	1.07
Social Inclusion	I47	6.52	0.81	5.42	1.53	6.96	1.11	6.38	1.48	7.38	0.95
Social Inclusion	I48	7.12	0.92	5.86	1.68	6.82	1.08	6.04	1.63	7.16	1.27
Social Inclusion	I49	7.00	1.11	6.40	1.33	7.04	1.11	6.56	1.76	7.54	1.09
Social Inclusion	I50	7.28	0.88	6.64	1.82	7.00	0.81	6.34	1.57	7.46	1.01
Social Inclusion	I51	6.34	0.75	5.46	1.56	6.48	1.00	5.66	1.61	7.00	1.01
Social Neutral	N1	5.78	1.09	4.54	1.69	5.20	0.83	4.28	1.54	5.26	1.12
Social Neutral	N2	5.32	0.82	4.08	1.66	5.10	0.84	4.26	1.70	5.22	0.86
Social Neutral	N3	4.16	1.10	3.74	1.48	4.68	0.82	3.86	1.60	4.72	0.81
Social Neutral	N4	5.00	1.20	3.76	1.64	5.18	0.90	4.22	1.73	5.16	1.02
Social Neutral	N5	4.64	0.80	3.98	1.39	5.42	0.84	4.04	1.71	5.20	0.88
Social Neutral	N6	4.62	0.78	3.86	1.54	5.28	0.86	4.26	1.76	5.16	0.74
Social Neutral	N7	4.42	0.97	3.50	1.53	5.28	0.88	4.02	1.84	5.30	0.86
Social Neutral	N8	5.10	0.97	3.92	1.61	5.42	1.09	4.32	1.86	5.42	1.03
Social Neutral	N9	5.34	0.82	4.34	1.60	5.28	0.78	4.06	1.58	5.36	1.05
Social Neutral	N10	4.14	1.16	3.66	1.59	5.18	0.77	4.04	1.64	5.34	1.08
Social Neutral	N11	3.92	1.03	3.44	1.50	5.44	0.84	4.08	1.87	5.54	1.15
Social Neutral	N12	4.04	1.09	3.36	1.77	5.50	0.97	4.44	1.62	5.44	1.09
Social Neutral	N13	4.16	1.13	3.54	1.59	5.04	0.67	4.24	1.51	5.18	0.90
Social Neutral	N14	4.62	0.75	4.02	1.70	4.86	1.03	4.22	1.65	5.26	0.99
Social Neutral	N15	4.16	1.30	4.70	1.67	5.10	0.84	4.00	1.69	5.08	0.80
Social Neutral	N16	4.30	0.95	3.90	1.52	5.24	0.92	3.92	1.68	5.26	1.10
Social Neutral	N17	5.28	1.01	4.46	1.45	4.80	0.78	4.48	1.59	4.88	1.10
Social Neutral	N18	4.76	0.87	4.24	1.35	5.10	0.74	4.10	1.46	5.14	0.76
Social Neutral	N19	5.48	0.95	3.80	1.87	5.60	1.03	3.84	1.89	5.34	0.92
Social Neutral	N20	5.60	1.18	3.86	1.69	5.42	0.97	4.02	1.77	5.36	1.16
Social Neutral	N21	5.26	1.07	3.72	1.65	5.46	0.95	3.64	1.64	5.26	0.97
Social Neutral	N22	4.28	1.11	4.14	1.51	5.46	1.01	4.38	1.71	5.48	1.02
Social Neutral	N23	5.58	0.93	4.36	1.55	5.56	0.95	4.18	1.52	5.78	1.25
Social Neutral	N24	4.46	0.99	3.64	1.59	5.28	1.05	4.52	1.52	5.38	1.12
Social Neutral	N25	4.78	1.06	4.06	1.62	5.34	0.80	3.90	1.64	5.36	1.01
Social Neutral	N26	4.42	1.01	4.62	1.52	4.70	0.95	4.06	1.52	4.56	1.25
Social Neutral	N27	5.38	0.92	4.52	1.49	5.44	1.01	4.48	1.76	5.42	1.01
Social Neutral	N28	4.68	1.10	3.98	1.52	5.58	1.23	4.14	1.71	5.18	0.90
Social Neutral	N29	4.76	0.85	4.00	1.51	5.20	0.90	4.38	1.55	5.18	0.98
Social Neutral	N30	6.08	1.10	5.42	1.90	5.78	1.11	5.04	2.00	5.32	0.79
Social Neutral	N31	5.14	0.95	4.26	1.54	5.68	0.98	4.84	1.74	5.44	0.88
Social Neutral	N32	4.48	1.39	4.02	1.67	5.18	0.77	4.06	1.54	5.06	0.98
Social Neutral	N33	5.48	1.05	3.98	1.60	5.52	0.86	4.12	1.53	5.48	0.95
Social Neutral	N34	3.88	1.26	3.88	1.77	4.44	1.30	4.04	1.55	4.46	1.07
Social Neutral	N35	5.16	1.00	4.14	1.82	5.46	1.05	4.24	1.39	5.20	0.93
Social Neutral	N36	4.90	0.93	3.98	1.53	5.24	0.59	4.14	1.58	5.18	0.77
Social Neutral	N37	5.06	0.89	4.04	1.81	5.28	0.93	4.74	1.59	5.24	0.63
Social Neutral	N38	3.94	1.20	4.78	1.97	5.18	0.83	4.06	1.74	5.32	0.87
Social Neutral	N39	5.70	1.00	4.72	1.69	5.14	0.76	3.98	1.66	5.24	0.96
Social Neutral	N40	4.22	1.08	3.90	1.62	5.04	0.64	4.08	1.50	5.22	0.91
Social Neutral	N41	5.14	1.01	4.28	1.79	5.18	0.52	4.10	1.63	5.28	0.67
Social Neutral	N42	4.60	1.18	3.70	1.57	5.04	0.78	4.26	1.56	5.14	0.93
Social Neutral	N43	4.56	1.05	4.52	1.85	4.84	0.65	4.16	1.62	5.00	0.86
Social Neutral	N44	4.72	0.95	3.88	1.55	5.24	0.63	4.18	1.55	5.24	0.77
Social Neutral	N45	4.82	0.98	3.80	1.60	4.58	1.14	4.26	1.40	4.50	0.97
Social Neutral	N46	5.02	0.98	4.12	1.77	5.36	0.78	3.98	1.65	5.38	0.95
Social Neutral	N47	5.36	1.05	3.66	1.60	5.58	1.01	4.20	1.85	5.36	0.90
Social Neutral	N48	4.80	0.95	3.86	1.43	4.96	0.49	3.98	1.60	5.08	0.88
Social Neutral	N49	4.70	0.97	3.90	1.36	5.28	0.90	4.22	1.74	5.12	0.87
Social Neutral	N50	4.62	0.78	3.80	1.31	5.38	0.78	4.28	1.72	5.32	0.74
Social Neutral	N51	4.90	0.89	4.18	1.65	5.10	0.84	4.10	1.56	5.28	1.07
Social Neutral	N52	4.84	0.96	4.02	1.56	5.18	0.75	3.96	1.71	5.12	0.80
Social Neutral	N53	3.60	1.09	3.76	1.60	4.78	0.65	3.88	1.55	4.70	0.86

Excel format can be downloaded from the provided website.
